# Selenium Dihalides Click Chemistry: Highly Efficient Stereoselective Addition to Alkynes and Evaluation of Glutathione Peroxidase-Like Activity of Bis(*E*-2-halovinyl) Selenides

**DOI:** 10.3390/molecules27031050

**Published:** 2022-02-03

**Authors:** Maxim V. Musalov, Vladimir A. Potapov, Arkady A. Maylyan, Alfiya G. Khabibulina, Sergey V. Zinchenko, Svetlana V. Amosova

**Affiliations:** A. E. Favorsky Irkutsk Institute of Chemistry, Siberian Division of The Russian Academy of Sciences, 1 Favorsky Str., 664033 Irkutsk, Russia; musalov_maxim@irioch.irk.ru (M.V.M.); maylyan@irioch.irk.ru (A.A.M.); almah@irioch.irk.ru (A.G.K.); svz@irioch.irk.ru (S.V.Z.); amosova@irioch.irk.ru (S.V.A.)

**Keywords:** alkynes, bis(2-bromovinyl) selenides, bis(2-chlorovinyl) selenides, selenium dibromide, selenium dichloride, stereoselective synthesis

## Abstract

Highly efficient stereoselective syntheses of novel bis(*E*-2-chlorovinyl) selenides and bis(*E*-2-bromovinyl) selenides in quantitative yields by reactions of selenium dichloride and dibromide with alkynes were developed. The reactions proceeded at room temperature as *anti*-addition giving products exclusively with (*E*)-stereochemistry. The glutathione peroxidase-like activity of the obtained products was estimated and compounds with high activity were found. The influence of substituents in the products on their glutathione peroxidase-like activity was discussed.

## 1. Introduction

The term “click chemistry” was coined by K. Barry Sharpless, in 1998 and was first fully described by Sharpless, Kolb, and Finn in 2001 [1]. They believe that click chemistry reactions must be wide in scope, give very high yields, and generate only inoffensive byproducts. The required process characteristics include simple reaction conditions, readily available starting materials and reagents, high selectivity and atom economy, and simple product isolation by non-chromatographic methods. The authors also included sulfenyl halides addition reactions to carbon–carbon multiple bonds to the click chemistry [1]. Although selenenyl halides additions were not mentioned, the chemical properties of these reagents are very similar to those of sulfenyl halides, but addition reactions of selenenyl halides often proceed with higher selectivity [2,3,4,5,6].

Organylselenenyl halides are widely used in modern organic synthesis [2,3,4,5,6]. In 2003, we first applied selenium dihalides in the synthesis of organoselenium compounds [7,8,9,10]. It is known that selenium dichloride and dibromide in solutions undergo disproportionation [11,12]. However, freshly prepared in situ from elemental selenium and sulfuryl chloride or bromine, these reagents can be successfully involved in further reactions [7,8,9,10]. Since then, the application of selenium dihalides in organic synthesis is intensively developing and makes it possible to obtain new classes of organoselenium compounds and selenium-containing heterocycles [13,14,15,16,17,18,19,20,21,22,23,24,25,26].

**Scheme 1:**
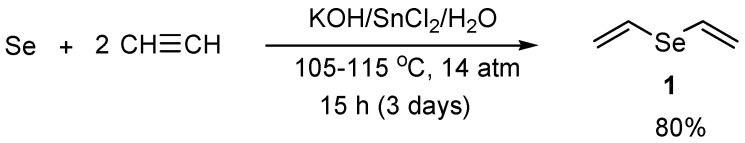
The synthesis of divinyl selenide (**1**) in 80% yield from elemental selenium and acetylene at 105–115 °C for 15 h (3 days, 5 h heating every day) under acetylene pressure in an autoclave.

The main methods for preparation of vinyl selenides are based on electrophilic addition of organylselenenyl halides to the triple bond as well as on nucleophilic addition of selenolate or selenide anions to acetylenes. Previously convenient methods for preparation of divinyl selenide and alkyl vinyl selenides from elemental selenium and acetylene were developed at this institute [27,28,29]. Unsubstituted divinyl selenide (**1**) was obtained in 80% yield from elemental selenium and acetylene in an aqueous solution of potassium hydroxide using tin dichloride as a reducing agent at 105–115 °C for 15 h (3 days, 5 h heating every day) under acetylene pressure (14 atm) in an autoclave (Scheme 1) [27,28].

Vinyl selenides are versatile intermediates and synthons for organic synthesis. A series of valuable products, including functionalized alkenes, ketones, (*Z*)-allyl alcohols, unsaturated aldehydes, and enyne derivatives, were obtained based on vinyl selenides [30,31,32,33,34,35,36,37,38].

The synthesis of resveratrol and its derivatives was realized in several stages from vinyl selenides [30]. Resveratrol and its methoxylated analogues are well known compounds due to the fact of their anti-inflammatory, anticancer, antibacterial and neuroprotective activity [30]. In addition, the cross-coupling reaction of vinyl selenides with terminal alkynes in the presence of a nickel/CuI catalyst at room temperature leading to (*Z*)- and (*E*)-enyne derivatives in good yields with retention of stereochemical configuration is very important [31]. Vinyl selenides, which exhibit antinociceptive [39], hepatoprotective [40], and antioxidant [41] activity, were found.

**Scheme 2:**
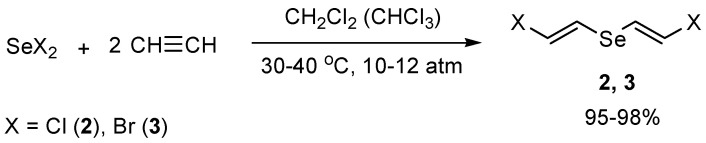
The synthesis of bis(*E*-2-chlorovinyl) selenide (2) and bis(*E*-2-bromovinyl) selenide (3) by electrophilic addition of selenium dihalides to acetylene.

Previously, we developed efficient synthesis of bis(*E*-2-chlorovinyl) selenide (**2**) and bis(*E*-2-bromovinyl) selenide (**3**) by electrophilic addition of selenium dihalides to acetylene [42] (Scheme 2). Some acetylene derivatives were also involved in this reaction [43,44,45,46].

**Scheme 3:**
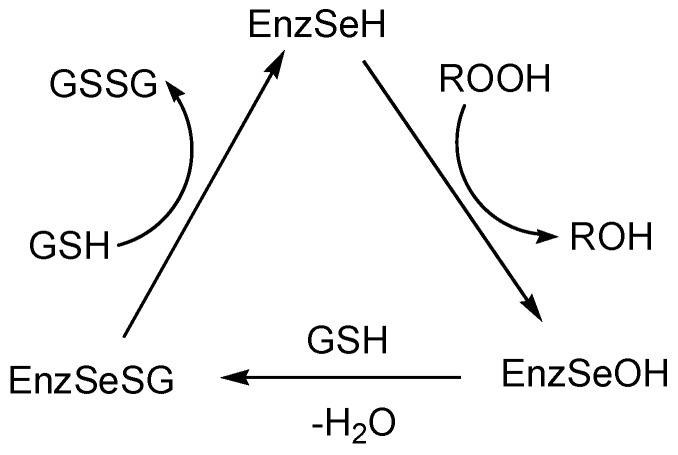
The catalytic cycle for the reduction of peroxides with the thiol glutathione (GSH) catalyzed by glutathione peroxidase (EnzSeH).

A number of functionalized organoselenium compounds, including selenium heterocycles, exhibit high glutathione peroxidase-like activity [14,47,48,49,50,51,52,53,54,55,56,57]. Glutathione peroxidase, which contains selenocysteine with the selenol function, is a selenium-containing enzyme that protects human cells by the catalytic reduction of peroxides with the thiol glutathione (the catalytic cycle is shown in Scheme 3) [55,56,57]. The selenol (EnzSeH) is oxidized by peroxides to the corresponding selenenic acid (EnzSeOH), which reacts with thiol glutathione (GSH) to form a selenenyl sulfide intermediate (EnzSeSG). The glutathione then regenerates the active form of the enzyme by the reaction with EnzSeSG to produce the oxidized glutathione GSSG. It was found that a number of organoselenium compounds can act as mimetics of glutathione peroxidase and play the role of catalysts in the reduction of peroxides with the thiols [14,47,48,49,50,51,52,53,54,55,56,57].

The organoselenium heterocyclic compound, ebselen, shows neuroprotective, anti-inflammatory, cytoprotective, and glutathione peroxidase-like properties [58,59,60,61,62,63]. Ebselen is used medicinally as an anti-inflammatory agent as well as for prevention of cardiovascular diseases and ischemic stroke. Furthermore, preliminary studies demonstrate that ebselen shows promising inhibitory activity against COVID-19 in cell-based assays [59]. The effect was attributed to irreversible inhibition of the main protease via a covalent bond formation with the thiol group of the active center’s cysteine (Cys-145).

Recently we developed the efficient regio- and stereoselective synthesis of the novel class of divinyl selenides, (*Z,Z*)-3,3′-selanediylbis(2-propenamides), based on the reaction of sodium selenide with 3-trimethylsilyl-2-propynamides [64]. Like ebselen, these products contain the amide function. The compounds with high glutathione peroxidase-like activity were found among (*Z,Z*)-3,3′-selanediylbis(2-propenamides) [64]. 

## 2. Results and Discussion

The goal of the present work was to develop efficient stereoselective syntheses of bis(2-chloroethyl) selenides and bis(2-bromoethyl) selenides by electrophilic addition of selenium dihalides to dialkylacetylenes (i.e., 2-butyne, 3-hexyne, 4-octyne, and 5-decyne) and the assessment of glutathione peroxidase-like activity of the obtained bis(2-halovinyl) selenides. Evaluation of the glutathione peroxidase-like activity of unsubstituted divinyl selenide **1**, bis(*E*-2-chlorovinyl) selenide **2,** and bis(*E*-2-bromovinyl) selenide **3** was planned, and their activity compared with that of the obtained bis(2-halovinyl) selenides. In addition, synthesis of bis(2-halovinyl) selenoxides by oxidation of corresponding bis(2-halovinyl) selenides was scheduled. The bis(2-halovinyl) selenoxides were supposed to be intermediates in the process of oxidation of dithiothreitol by *tert*-butyl hydroperoxide on the assessment of glutathione peroxidase-like activity of the obtained bis(2-halovinyl) selenides. 

**Scheme 4:**
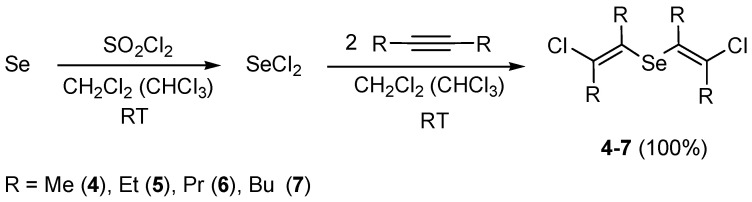
The synthesis of bis(2-chlorovinyl) selenides **4**–**7** by addition of selenium dichloride to dialkylacetylenes (i.e., 2-butyne, 3-hexyne, 4-octyne, and 5-decyne).

The efficient stereoselective synthesis of bis(*E*-2-chlorovinyl) selenides (**4**–**7**) in quantitative yields was developed by electrophilic addition of selenium dichloride to dialkylacetylenes (i.e., 2-butyne, 3-hexyne, 4-octyne, and 5-decyne). The reaction proceeded in methylene chloride or chloroform at room temperature in a stereoselective fashion as *anti*-addition producing products exclusively with (*E)*-stereochemistry (Scheme 4). 

Selenium dichloride was freshly prepared in situ from elemental selenium and sulfuryl chloride and immediately involved in further reactions (Scheme 4). Removing the solvent from the reaction mixture followed by drying in vacuum led to pure products **4**–**7** in quantitative yields.

**Scheme 5:**
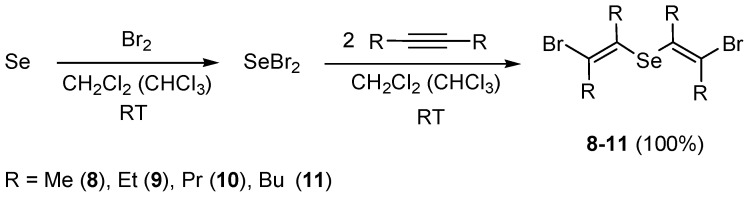
The synthesis of bis(2-bromovinyl) selenides **8**–**11** by addition of selenium dibromide to dialkylacetylenes (i.e., 2-butyne, 3-hexyne, 4-octyne, and 5-decyne).

The reaction of selenium dibromide with 2-butyne, 3-hexyne, 4-octyne, and 5-decyne was realized in a similar manner. Selenium dibromide was produced by mixing elemental selenium and a solution of bromine in methylene chloride or chloroform. After dissolution of the selenium, the obtained solution of selenium dibromide was added dropwise to a solution of dialkylacetylene in methylene chloride or chloroform, and the reaction mixture was stirred for 1–3 h at room temperature. After removing the solvent from the reaction mixture by a rotary evaporator, the residue was dried in vacuum giving bis(*E*-2-bromovinyl) selenides **8**–**11** (quantitative yields), which did not require additional purification. The reaction proceeded in a stereoselective mode as *anti*-addition affording products only with (*E*)-configuration (Scheme 5). 

**Scheme 6:**
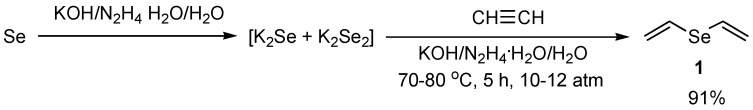
The synthesis of divinyl selenide **1** in 91% yield from elemental selenium and acetylene at 70–80 °C for 5 h under acetylene pressure in an autoclave.

Divinyl selenide **1** was obtained by a modified procedure in 91% yield from elemental selenium and acetylene in an aqueous solution of potassium hydroxide and hydrazine hydrate at 70–80 °C for 5 h under acetylene pressure in an autoclave (Scheme 6). 

The isolation of the target product did not require organic solvents for extraction: the organic phase was simply separated from the reaction mixture by a separatory funnel. This method of carrying out the reaction in water without using organic solvents can be considered as a “green chemistry method”. This procedure is superior to the earlier method [27,28] in the yield of the target product, the duration (5 h instead of 15 h, 3 days) and the temperature of the process (70–80 °C instead of 105–115 °C). 

Selenide **1** was used for the glutathione peroxidase-like activity studies, and its activity was compared with that of bis(2-halovinyl) selenides **4**–**11**. 

**Scheme 7:**
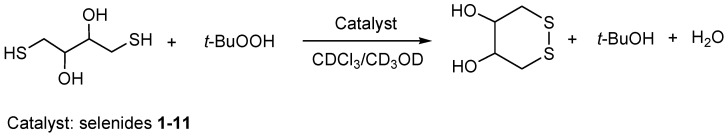
The model reaction of dithiothreitol oxidation by *tert*-butyl hydroperoxide in the presence of a catalytic number of synthesized compounds as catalysts (10 mol%).

The glutathione peroxidase-like activity of the obtained products was estimated using the model reaction of dithiothreitol oxidation by *tert*-butyl hydroperoxide (Scheme 7) in the presence of a catalytic number of synthesized compounds as a catalysts (10 mol%) [14,47,48,49,50,51]. The progress of this reaction was monitored by ^1^H NMR spectroscopy at room temperature (dithiothreitol, 0.07 mmol; *tert*-butyl hydroperoxide, 0.07 mmol; tested product, 0.007 mmol; deuterochloroform/CD_3_CD = 95/5, 0.5 mL). The control experiment was conducted under the same reaction conditions but in the absence of the catalyst. 

**Figure 1:**
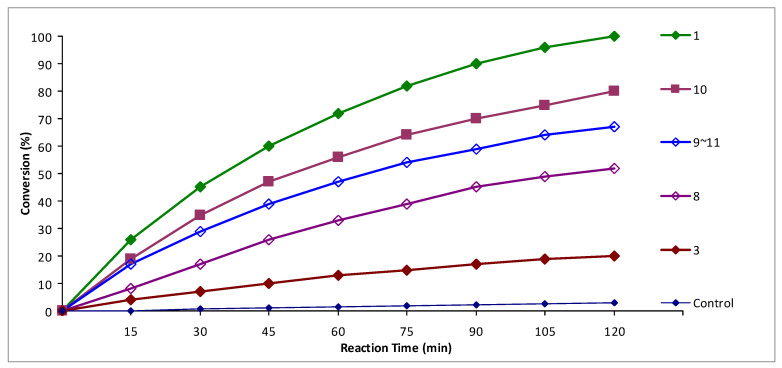
The assessment of glutathione peroxidase-like activity of divinyl selenide **1** and bis(2-bromovinyl) selenides **3**, **8**–**11** by ^1^H-NMR monitoring.

**Figure 2:**
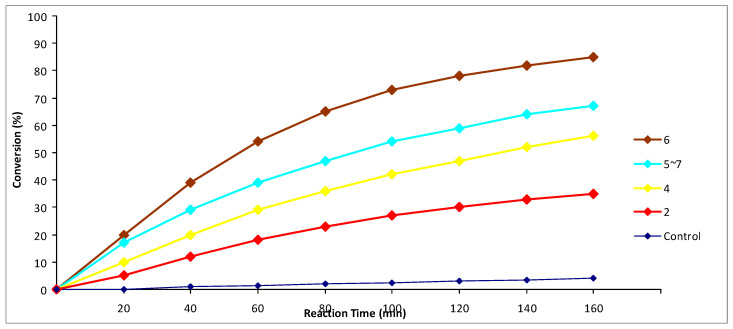
The assessment of glutathione peroxidase-like activity of bis(2-chlorovinyl) selenides **2**, **4**–**7** by ^1^H-NMR monitoring.

It was found that unsubstituted divinyl selenide **1** showed the best activity among the tested selenides (Figure 1). The activity of bis(2-bromovinyl) selenides, in general, exceeds the activity of bis(2-chlorovinyl) selenides (Figure 1 and Figure 2). This trend can be explained in terms of electron density on the selenium atom in divinyl selenide, bis(2-chlorovinyl) selenides, and bis(2-bromovinyl) selenides. We suppose that the electron density on the selenium atom and the presence of electron-withdrawing groups, which are in conjugation with double bonds and an unshared electron pair of the selenium atom, can affect redox processes and manifestation of the glutathione peroxidase-like activity. Since bromine and especially chlorine are electronegative atoms, they can decrease the electron density on the selenium atom in bis(2-chlorovinyl) selenides and bis(2-bromovinyl) selenides. The chlorine atom was superior to the bromine atom in electronegativity and the glutathione peroxidase-like activity of bis(2-bromovinyl) selenides exceeded the activity of chloro-containing selenides (Figure 1 and Figure 2). Unsubstituted divinyl selenide does not have electronegative heteroatoms, and it showed the best activity among the tested selenides.

Another trend, which can be seen based on the obtained data (Figure 1 and Figure 2), was the increase in glutathione peroxidase-like activity with the increasing length of the carbon skeleton in tested molecules. However, in the case of 5-decyne derivatives **7** and **11**, XC(Bu) = C(Bu)SeC(Bu) = C(Bu)X, the activity decreased and was lower than the activity of 4-octyne derivatives **6** and **10**, XC(Pr) = C(Pr)SeC(Pr) = C(Pr)X (Figure 1 and Figure 2). We assume that the steric factor begins to manifest itself in the latter case, and the selenium atom in the 5-decyne derivative becomes sterically less accessible for redox processes.

It is worth noting that this is the first example of glutathione peroxidase-like activity assessment of divinyl selenide and bis(2-halovinyl) selenides, which do not contain additional heteroatoms. 

Bis(2-chlorovinyl) and bis(2-bromovinyl) selenoxides were supposed to be intermediates in the catalytic process of oxidation of dithiothreitol by *tert*-butyl hydroperoxide on the assessment of glutathione peroxidase-like activity of the corresponding selenides. 

**Scheme 8:**
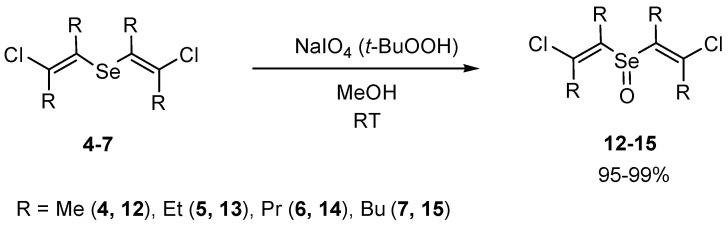
The synthesis of bis(2-chlorovinyl) selenoxides **12**–**15** by oxidation of selenides **4**–**7**.

**Scheme 9:**
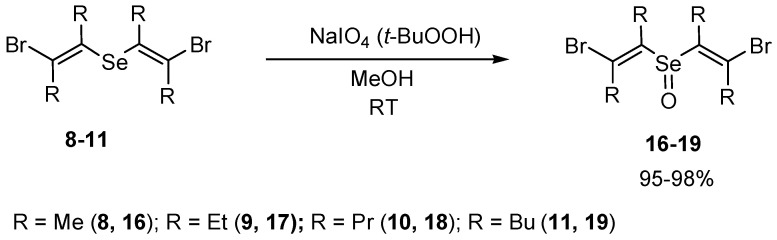
The synthesis of bis(2-bromovinyl) selenoxides **16**–**19** by oxidation of selenides **8**–**11**.

The efficient syntheses of novel families of bis(2-chlorovinyl) selenoxides **12**–**15** (Scheme 8) and bis(2-bromovinyl) selenoxides **16**–**19** (Scheme 9) in 95–99% yields by oxidation of corresponding selenides with sodium metaperiodate or *tert*-butyl hydroperoxide were developed. The application of sodium metaperiodate for the oxidation of the selenides made it possible to obtain cleaner products in comparison with the use of *tert*-butyl hydroperoxide. 

**Scheme 10:**
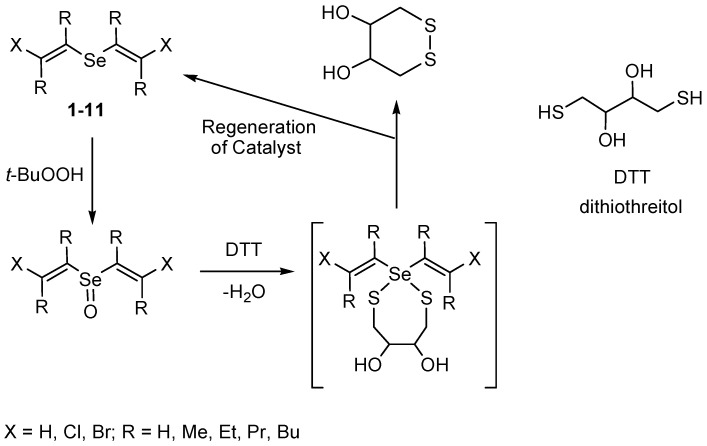
A proposed catalytic cycle to explain the catalytic effect of the obtained compounds.

As a rule, compounds with a sulfur−selenium bond are considered as intermediates in the oxidation reactions of thiols by peroxides catalyzed by organoselenium compounds [14,47,48,49,50,51]. In our case, the following scheme can be proposed to explain the catalytic effect of the obtained compounds (Scheme 10). The reaction of the formed selenoxides with dithiothreitol is assumed to lead to the heterocyclic intermediate, which undergoes conversion to the oxidized form of dithiothreitol with regeneration of the catalyst. 

Thus, stereoselective syntheses of novel bis(*E*-2-chlorovinyl)selenides and bis(*E*-2-bromovinyl)selenides in quantitative yields by electrophilic addition reactions of selenium dichloride and selenium dibromide to dialkylacetylenes were developed. The glutathione peroxidase-like activity of the obtained products was estimated and compounds with high activity were found. 

## 3. Experimental Section

### 3.1. General Information

The ^1^H (400.1 MHz) and ^13^C (100.6 MHz) NMR spectra (see Supplementary Materials) were recorded on a Bruker DPX-400 spectrometer (Bruker BioSpin GmbH, Rheinstetten, Germany) in CDCl_3_ solutions and referred to the residual solvent peaks of CDCl_3_ (δ = 7.27 and 77.16 ppm in ^1^H- and ^13^C-NMR, respectively). Elemental analysis was performed on a Thermo Scientific Flash 2000 Elemental Analyzer (Thermo Fisher Scientific Inc., Milan, Italy). The organic solvents were dried and distilled according to standard procedures.

### 3.2. Synthesis of Selenides 

*Divinyl selenide* (**1**). Hydrazine hydrate (7 mL) and a cold solution of KOH (85%, 9 g, 0.136 mol) in water (40 mL) were added to selenium powder (7.9 g, 0.1 mol) and the mixture was stirred overnight. The next day, the resulting mixture was heated (70–80 °C) in a 1 L rotating autoclave under the pressure of acetylene (10–12 atm) for 5 h. The lower organic phase was separated from the reaction mixture by a separatory funnel, dried over Na_2_SO_4_ and distilled at reduced pressure (85–95 mm Hg) giving divinyl selenide [27,28] (12.1 g, 91% yield), bp 45–47 °C (90–92 mm Hg).

*Bis(E-2-chloro-1-methyl-1-propenyl) selenide* (**4**). A solution of selenium dichloride (1 mmol) in methylene chloride (2 mL) was added dropwise to a solution of 2-butyne (108 mg, 2 mmol) in methylene chloride (18 mL). The mixture was stirred for 1 h at room temperature. The solvent was removed by a rotary evaporator and the residue was dried in vacuum giving compound **4** (258 mg) as a light yellow oil in quantitative yield. 

^1^H-NMR (400 MHz): 2.49 (s, 6H, CH_3_), 2.54 (s, 6H, CH_3_). ^13^C-NMR (100 MHz): 23.7 (CH_3_), 26.3 (CH_3_), 121.8 (CSe, J_C-Se_ 106.4 Hz), 130.7 (CCl). IR (KBr): λ = 2950, 2916, 2848, 1621 (C=C), 1435 cm^−1^.

Anal. calcd. for C_8_H_12_Cl_2_Se (258.05): C 37.24, H 4.69, Cl 27.48, Se 30.60%. Found: C 37.51, H 4.78, Cl 27.23, Se 30.38%.

*Bis(E-2-chloro-1-ethyl-1-butenyl) selenide* (**5**) was obtained under the same conditions as compound **4** from selenium dichloride and 3-hexyne as a light yellow oil in quantitative yield. 

^1^H-NMR (400 MHz): 1.16 (t, 6H, CH_3_), 1.23 (t, 6H, CH_3_), 2,48 (q, 4H, CH_2_), 2.84 (q, 4H, CH_2_). ^13^C-NMR (100 MHz): 12.7 (CH_3_), 13.3 (CH_3_), 30.0 (CH_2_), 33.1 (CH_2_), 128.6 (CSe, J_C-Se_ 106.8 Hz), 137.4 (CCl). IR (KBr): λ = 2971, 2932, 2873, 1606 (C=C), 1458 cm^−1^.

Anal. calcd for C_12_H_20_Cl_2_Se (314.15): C 45.88, H 6.42, Cl 22.57, Se 25.13%. Found: C 46.13, H 6.56, Cl 22.45, Se 24.80%.

*Bis(E-2-chloro-1-propyl-1-pentenyl) selenide* (**6**) was obtained under the same conditions as compound **4** but during 2 h from selenium dichloride and 4-octyne as a light yellow oil in quantitative yield. 

^1^H-NMR (400 MHz): 1.00 (t, 6H, CH_3_), 1.02 (t, 6H, CH_3_), 1.58–1.73 (m, 8H, CH_2_CH_2_CSe, CH_2_CH_2_CCl) 2.38–2.45 (m, 4H, CH_2_), 2.76–2.81 (m, 4H, CH_2_). ^13^C-NMR (100 MHz): 13.2 (CH_3_), 13.7 (CH_3_), 21.3 (CH_2_), 21.4 (CH_2_), 37.8 (CH_2_), 40.8 (CH_2_), 128.3 (CSe, J_C-Se_ 106.4 Hz), 135.9 (CCl). IR (KBr): λ = 2961, 2931, 2871, 1606 (C=C), 1461 cm^−1^.

Anal. calcd. for C_16_H_28_Cl_2_Se (370.26): C 51.90, H 7.62, Cl 19.15, Se 21.33%. Found: C 51.79, H 7.71, Cl 18.86, Se 21.50%.

*Bis(E-2-chloro-1-butyl-1-hexenyl) selenide* (**7**) was obtained under the same conditions as compound **4** but for 2 h from selenium dichloride and 5-decyne as a light yellow oil in quantitative yield. 

^1^H-NMR (400 MHz): 0.91 (t, 6H, CH_3_), 0.94 (t, 6H, CH_3_), 1.28–1.40 (m, 8H, CH_3_CH_2_), 1.44–1.59 (m, 8H, CH_2_CH_2_CSe, CH_2_CH_2_CCl) 2.34–2,40 (m, 4H, CH_2_), 2.70–2.76 (m, 4H, CH_2_). ^13^C-NMR (100 MHz): 14.2 (CH_3_), 22.1 (CH_2_), 22.5 (CH_2_), 30.1 (CH_2_), 30.4 (CH_2_), 35.8 (CH_2_), 39.0 (CH_2_), 128.1 (CSe, J_C-Se_ 107.0 Hz), 135.9 (CCl). IR (KBr): λ = 2957, 2928, 2860, 1607 (C=C), 1463 cm^−1^.

Anal. calcd. for C_20_H_36_Cl_2_Se (426.36): C 56.34, H 8.51, Cl 16.63, Se 18.52%. Found: C 56.06, H 8.75, Cl 16.42, Se 18.86%.

*Bis(E-2-bromo-1-methyl-1-propenyl) selenide* (**8**). A solution of selenium dibromide (1 mmol) in methylene chloride (2 mL) was added dropwise to a solution of 2-butyne (108 mg, 2 mmol) in methylene chloride (18 mL). The mixture was stirred for 1 h at room temperature. The solvent was removed by a rotary evaporator and the residue was dried in vacuum giving compound **8** (347 mg) as a light yellow oil in quantitative yield. 

^1^H-NMR (400 MHz): 2.19 (s, 6H, CH_3_), 2.54 (s, 6H, CH_3_). ^13^C-NMR (100 MHz): 27.1 (CH_3_), 29.2 (CH_3_), 121.9 (CBr), 123.7 (CSe, J_C-Se_ 107.5 Hz). IR (KBr): λ = 2949, 2914, 2847, 1621 (C=C), 1433 cm^−1^.

Anal. calcd. for C_8_H_12_Br_2_Se (346.95): C 27.69, H 3.49, Br 46.06, Se 22.76%. Found: C 27.91, H 3.30, Br 45.83, Se 23.02%.

*Bis(E-2-bromo-1-ethyl-1-butenyl) selenide* (**9**) was obtained under the same conditions as compound **8** but for 2 h from selenium dibromide and 3-hexyne as a light yellow oil in quantitative yield. 

^1^H-NMR (400 MHz): 1.05 (t, 6H, CH_3_), 1.10 (t, 6H, CH_3_), 2,39 (q, 4H, CH_2_), 2.83 (q, 4H, CH_2_). ^13^C-NMR (100 MHz): 11.8 (CH_3_), 13.2 (CH_3_), 32.4 (CH_2_), 34.9 (CH_2_), 129.3 CBr), 129.7 (CSe, J_C-Se_ 108 Hz). IR (KBr): λ = 2970, 2932, 2873, 1607 (C=C), 1456 cm^−1^.

Anal. calcd. for C_16_H_28_Br_2_SeO (403.06): C 35.76, H 5.00, Br 39.65, Se 19.59%. Found: C 36.03, H 5.17, Br 39.38, Se 19.34%.

*Bis(E-2-bromo-1-propyl-1-pentenyl) selenide* (**10**) was obtained under the same conditions as compound **8** but for 2 h from selenium dibromide and 4-octyne as a light yellow oil in quantitative yield. 

^1^H-NMR (400 MHz): 0.83–0.91 (m, 12H, CH_3_), 1.37–1.68 (m, 8H, CH_2_CH_2_CSe, CH_2_CH_2_CBr) 2.46–2.58 (m, 8H, CH_2_CSe, CH_2_CBr). ^13^C-NMR (100 MHz): 13.6 (CH_3_), 14.2 (CH_3_), 21.6 (CH_2_), 22.4 (CH_2_), 41.3 (CH_2_), 43.5 (CH_2_), 128.8 (CSe J_C-Se_ 108 Hz), 130.2 (CCl). IR (KBr): λ = 2960, 2930, 2871, 1604 (C=C), 1460 cm^−1^.

Anal. calcd. for C_16_H_28_Br_2_SeO (459.16): C 41.85, H 6.15, Br 34.80, Se 17.20%. Found: C 42.03, H 6.27, Br 35.05, Se 16.99%.

*Bis(E-2-bromo-1-butyl-1-hexenyl) selenide* (**11**) was obtained under the same conditions as compound **8** but for 3 h from selenium dibromide and 5-decyne as a light yellow oil in quantitative yield. 

^1^H-NMR (400 MHz): 0.92 (t, 6H, CH_3_), 0.95 (t, 6H, CH_3_), 1.30–1.39 (m, 8H, CH_3_CH_2_), 1.48–1.56 (m, 8H, CH_2_CH_2_CSe, CH_2_CH_2_CBr) 2.35–2,42 (m, 4H, CH_2_), 2.80–2.87 (m, 4H, CH_2_). ^13^C-NMR (100 MHz): 14.1 (CH_3_), 14.1 (CH_3_), 21.9 (CH_2_), 22.4 (CH_2_), 30.0 (CH_2_), 31.0 (CH_2_), 38.9 (CH_2_), 41.3 (CH_2_), 128.5 (CBr), 129.7 (CSe, J_C-Se_ 108.0 Hz). IR (KBr): λ = 2956, 2926, 2859, 1606 (C=C), 1462 cm^−1^.

Anal. calcd. for C_20_H_36_Br_2_Se (515.27): C 46.62, H 7.04, Br 31.01, Se 15.32%. Found: C 46.34, H 7.00, Br 30.89, Se 15.45%.

### 3.3. Synthesis of Selenoxides

*Bis(E-2-chloro-1-methyl-1-propenyl) selenoxide* (**12**). Sodium metaperiodate (257 mg, 1.2 mmol) was added to a solution of selenide **4** (258 mg, 1 mmol) in absolute methanol (15 mL). The mixture was stirred overnight (16 h) at room temperature. The mixture was filtered. The solvent was removed by a rotary evaporator from the filtrate and the residue was dried in vacuum giving compound **12** (266 mg) as a light yellow oil in 97% yield. 

^1^H-NMR (400 MHz): 1.99 (s, 3H, CH_3_), 2.00 (s, 3H, CH_3_), 2.31 (s, 3H, CH_3_), 2.32 (s, 3H, CH_3_). ^13^C-NMR (100 MHz): 12.7 (CH_3_) 24.4 (CH_3_), 135.1 (CSe, J_C-Se_ 106.4 Hz), 135.6 (CCl). IR (KBr): λ = 2958, 2919, 2852, 1640 (C=C), 1441, 918, 840 (Se=O) cm^−1^.

Anal. calcd. for C_8_H_12_Cl_2_SeO (274.05): C 35.06, H 4.41, Cl 25.87, Se 28.81%. Found: C 35.34, H 4.57, Cl 25.58, Se 28.61%.

*Bis(E-2-chloro-1-ethyl-1-butenyl) selenoxide* (**13**) was obtained under the same conditions as compound **12** by oxidation of selenide **5** as a light yellow oil in 95% yield. 

^1^H-NMR (400 MHz): 1.09 (t, 6H, CH_3_), 1.19 (t, 6H, CH_3_), 251–2.75 (m, 8H, CH_2_CSe, CH_2_CCl). ^13^C-NMR (100 MHz): 12.4 (CH_3_), 13.2 (CH_3_), 21.1 (CH_2_), 30.1 (CH_2_), 140.1 (CSe, J_C-Se_ 119.4 Hz), 142.2 (CCl). IR (KBr): λ = 2975, 2937, 2877, 1623 (C=C), 1458, 913, 842 (Se=O) cm^−1^.

Anal. calcd. for C_12_H_20_Cl_2_SeO (330.15): C 43.66, H 6.11, Cl 21.48, Se 23.92%. Found: C 43.44, H 5.98, Cl 21.22, Se 24.18%.

*Bis(E-2-chloro-1-propyl-1-pentenyl) selenoxide* (**14**) was obtained under the same conditions as compound **12** by oxidation of selenide **6** as a light yellow oil in 96% yield.

^1^H-NMR (400 MHz): 0.81–0.87 (m, 12H, CH_3_), 1.33–1.65 (m, 8H, CH_2_CH_2_CSe, CH_2_CH_2_CCl) 234–2.56 (m, 8H, CH_2_CSe, CH_2_CCl). ^13^C-NMR (100 MHz): 13.0 (CH_3_), 14.0 (CH_3_), 20.9 (CH_2_), 22.0 (CH_2_), 29.7 (CH_2_), 39.0 (CH_2_), 139.8 (CSe, J_C-Se_ 118.6 Hz), 140.8 (CCl). IR (KBr): λ = 2963, 2932, 2873, 1621 (C=C), 1461, 911, 841 (Se=O) cm^−1^.

Anal. calcd. for C_16_H_28_Cl_2_SeO (386.26): C 49.75, H 7.31, Cl 18.36, Se 20.44%. Found: C 50.03, H 7.45, Cl 18.50, Se 20.26%.

*Bis(E-2-chloro-1-butyl-1-hexenyl) selenoxide* (**15**) was obtained under the same conditions as compound **12** by oxidation of selenide **7** as a light yellow oil in 99% yield. 

^1^H-NMR (400 MHz): 0.76–0.82 (m, 12H, CH_3_), 1.19−1.58 (m, 16H, CH_3_CH_2_, CH_2_CH_2_CSe, CH_2_CH_2_CCl) 2.34–2,52 (m, 8H, CH_2_CSe, CH_2_CCl). ^13^C-NMR (100 MHz): 13.5 (CH_3_), 13.6 (CH_3_), 21.8 (CH_2_), 22.7 (CH_2_), 27.5 (CH_2_), 29.7 (CH_2_), 30.6 (CH_2_), 37.0 (CH_2_), 139.8 (CSe, J_C-Se_ 120.8 Hz), 140.6 (CCl). IR (KBr): λ = 2959, 2931, 2872, 1620 (C=C), 1464, 909, 837 (Se=O) cm^−1^.

Anal. calcd. for C_20_H_36_Cl_2_SeO (442.36): C 54.30, H 8.20, Cl 16.03, Se 17.85%. Found: C 54.07, H 8.31, Cl 15.92, Se 18.09%.

*Bis(E-2-bromo-1-methyl-1-propenyl) selenoxide* (**16**) was obtained under the same conditions as compound **12** by oxidation of selenide **8** as a light yellow oil in 95% yield. 

^1^H-NMR (400 MHz): 2.02 (s, 3H, CH_3_), 2.03 (s, 3H, CH_3_), 2.51 (s, 3H, CH_3_), 2.51 (s, 3H, CH_3_). ^13^C-NMR (100 MHz): 15.77 (CH_3_), 27.0 (CH_3_), 126.4 (CBr), 137.4 (CSe, J_C-Se_ 107.5 Hz). IR (KBr): λ = 2957, 2918, 2852, 1639 (C=C), 1440, 916, 840 (Se=O) cm^−1^.

Anal. calcd. for C_8_H_12_Br_2_SeO (362.95): C 26.47, H 3.33, Br 44.03, Se 21.76%. Found: C 26.71, H 3.48, Br 43.88, Se 21.96%.

*Bis(E-2-bromo-1-ethyl-1-butenyl) selenoxide* (**17**) was obtained under the same conditions as compound **12** by oxidation of selenide **9** as a light yellow oil in 96% yield. 

^1^H-NMR (400 MHz): 1.09 (t, 6H, CH_3_), 1.19 (t, 6H, CH_3_), 2.53–2.72 (m, 8H, CH_2_CSe, CH_2_CBr). ^13^C-NMR (100 MHz): 12.9 (CH_3_), 13.2 (CH_3_), 23.5 (CH_2_), 37.8 (CH_2_), 134.7 (CBr), 141.7 (CSe, J_C-Se_ 120.6 Hz). IR (KBr): λ = 2974, 2935, 2876, 1621 (C=C), 1457, 915, 843 (Se=O) cm^−1^.

Anal. calcd. for C_12_H_20_Br_2_SeO (419.05): C 34.39, H 4.81, Br 38.14, Se 18.84%. Found: C 34.15, H 4.84, Br 37.88, Se 19.12%.

*Bis(E-2-bromo-1-propyl-1-pentenyl) selenoxide* (**18**) was obtained under the same conditions as compound **12** by oxidation of selenide **10** as a light yellow oil in 97% yield.

^1^H-NMR (400 MHz): 0.94–1.00 (m, 12H, CH_3_), 1.45–1.79 (m, 8H, CH_2_CH_2_CSe, CH_2_CH_2_CBr) 2.46–2.55 (m, 4H, CH_2_CSe, CH_2_CBr). ^13^C-NMR (100 MHz): 12.9 (CH_3_), 14.0 (CH_3_), 21.8 (CH_2_), 21.9 (CH_2_), 32.1 (CH_2_), 40.9 (CH_2_), 133.3 (CBr), 141.8 (CSe, J_C-Se_ 122.0 Hz). IR (KBr): λ = 2962, 2931, 2872, 1620 (C=C), 1460, 912, 840 (Se=O) cm^−1^.

Anal. calcd. for C_16_H_28_Br_2_SeO (475.16): C 40.44, H 5.94, Br 33.63, Se 16.62%. Found: C 40.21, H 6.13, Br 33.47, Se 16.88%.

*Bis(E-2-chloro-1-butyl-1-hexenyl) selenoxide* (**19**) was obtained under the same conditions as compound **12** by oxidation of selenide **11** as a light yellow oil in 98% yield. 

^1^H-NMR (400 MHz): 0.81–0.90 (m, 12H, CH_3_), 1.22–1.55 (m, 16H, CH_3_CH_2_, CH_2_CH_2_CSe, CH_2_CH_2_CBr) 2.34–2.71 (m, 8H, CH_2_CSe, CH_2_CBr). ^13^C-NMR (100 MHz): 13.6 (CH_3_), 13.8 (CH_3_), 21.9 (CH_2_), 22.8 (CH_2_), 30.3 (CH_2_), 30.6 (CH_2_), 30.7 (CH_2_), 39.3 (CH_2_), 134.1 (CBr), 141.0 (CSe, J_C-Se_ 121.7 Hz). IR (KBr): λ = 2959, 2930, 2872, 1618 (C=C), 1463, 909, 837 (Se=O) cm^−1^.

Anal. calcd. for C_20_H_36_Br_2_SeO (531.27): C 45.22, H 6.83, Br 30.08, Se 14.86%. Found: C 44.87, H 6.93, Br 29.84, Se 15.12%.

## 4. Conclusions

The stereoselective syntheses of novel bis(*E*-2-chlorovinyl) and bis(*E*-2-bromovinyl) selenides in quantitative yields by electrophilic addition reactions of selenium dichloride and selenium dibromide to dialkylacetylenes were developed. The reactions proceeded as *anti*-addition producing products exclusively with (*E*)-stereochemistry. The reactions can be regarded as selenium dihalides click chemistry due to the quantitative yields and 100% stereoselectivity.

The glutathione peroxidase-like activity of the obtained products was estimated and compounds with high activity were found. It was revealed that unsubstituted divinyl selenide showed the best activity among the tested selenides. The activity of bis(2-bromovinyl) selenides, in general, exceeds the activity of bis(2-chlorovinyl) selenides. This trend was explained in terms of electron density on the selenium atom in divinyl selenides. The chlorine atom was superior to the bromine atom in electronegativity, and the glutathione peroxidase-like activity of bis(2-bromovinyl) selenides exceeded the activity of chloro-containing selenides. Unsubstituted divinyl selenide does not have electronegative heteroatoms, and it showed the best activity among the tested selenides.

Another observed trend was the increase in the glutathione peroxidase-like activity with the increasing length of the carbon skeleton in the tested molecules from XCH = CHSeCH = CHX to XC(Pr) = C(Pr)SeC(Pr) = C(Pr)X (X = Cl, Br). However, in the case of 5-decyne derivatives **7** and **11**, the activity decreased and was lower than the activity of 4-octyne derivatives **6** and **10**. The steric factor was assumed to manifest itself in the latter case, and the selenium atom in the 5-decyne derivatives became sterically less accessible for redox processes.

The synthesis of novel families of bis(2-chlorovinyl) selenoxides and bis(2-bromovinyl) selenoxides in 95–99% yields by oxidation of corresponding bis(2-halorovinyl) selenides with sodium metaperiodate was developed. The selenoxides were supposed to be intermediates in the catalytic process of oxidation of dithiothreitol by *tert*-butyl hydroperoxide. 

## Data Availability

Not applicable.

## Supplementary Materials

The following are available online, the NMR spectra of the obtained compounds.

## Sample Availability

Samples of the compounds are not available from the authors.
