# Supplementary material for: Selenium Dihalides Click Chemistry: Highly Efficient Stereoselective Addition to Alkynes and Evaluation of Glutathione Peroxidase-Like Activity of Bis(E-2-halovinyl) Selenides
Source: Molecules. 2022 Feb 3;27(3):1050. doi: 10.3390/molecules27031050 (PMC8838769; doi:10.3390/molecules27031050)
Supplement: Supplementary file 1 [file molecules-27-01050-s001.zip › molecules-1560421-supplementary.pdf]

Supplementary Materials

# Selenium Dihalides Click Chemistry: Highly Efficient Stereoselective Addition to Alkynes and Evaluation of Glutathione Peroxidase-Like Activity of Bis(***E***-2-halovinyl) Selenides

Maxim V. Musalov, Vladimir A. Potapov,* Arkady A. Maylyan, Alfiya G. Khabibulina, Sergey V. Zinchenko and Svetlana V. Amosova

[1] A. E. Favorsky Irkutsk Institute of Chemistry, Siberian Division of The Russian Academy of Sciences, 1 Favorsky Str., Irkutsk 664033, Russia; musalov_maxim@irioch.irk.ru (M.V.M.); maylyan@irioch.irk.ru (A.A.M.); almah@irioch.irk.ru (A.G.K.); svz@irioch.irk.ru (S.V.Z.); amosova@irioch.irk.ru (S.V.A.)
Correspondence: v.a.potapov@mail.ru

## Table of Contents

Experimental (General Information) 2

Examples of $^{1}H$ and $^{13}C$-NMR Spectra 3-17

Experimental (General Information)

$^{1}H$ (400.1 MHz) and $^{13}C$ (100.6 MHz) NMR spectra were recorded on a Bruker DPX-400 spectrometer (Bruker BioSpin GmbH, Rheinstetten, Germany) in $CDCl_3$ and referred to the residual solvent peaks of $CDCl_3$ ($\delta$ = 7.27 and 77.16 ppm in $^{1}H$- and $^{13}C$-NMR, respectively).

# Examples of $^1H$ and $^{13}C$-NMR Spectra

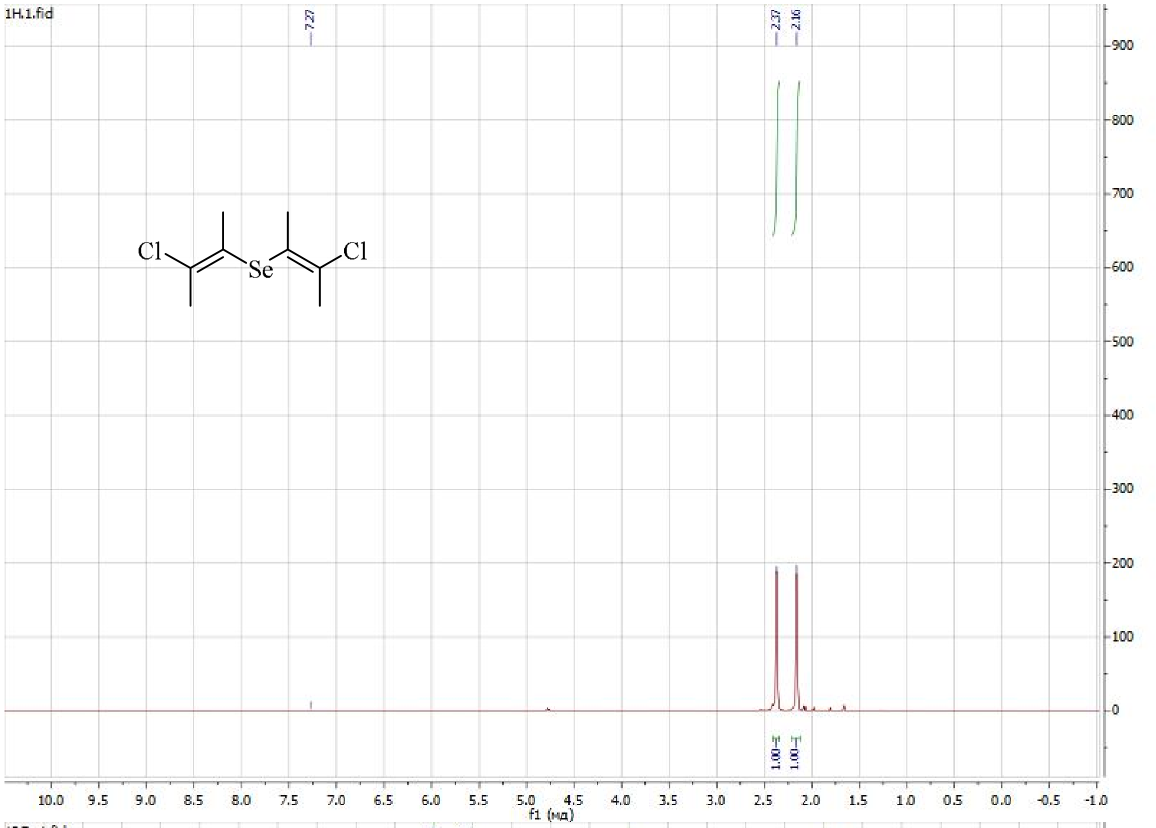


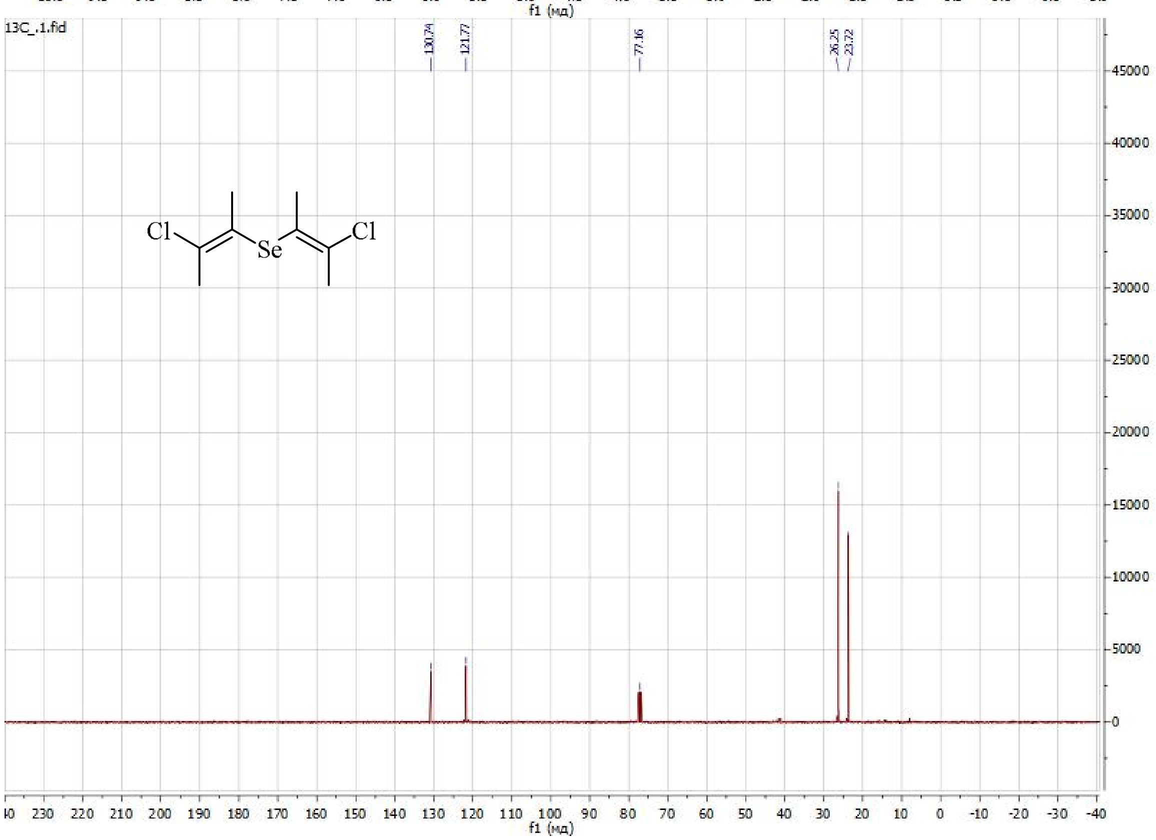


**$^1H$- and $^{13}C$-NMR spectra of compound 4**

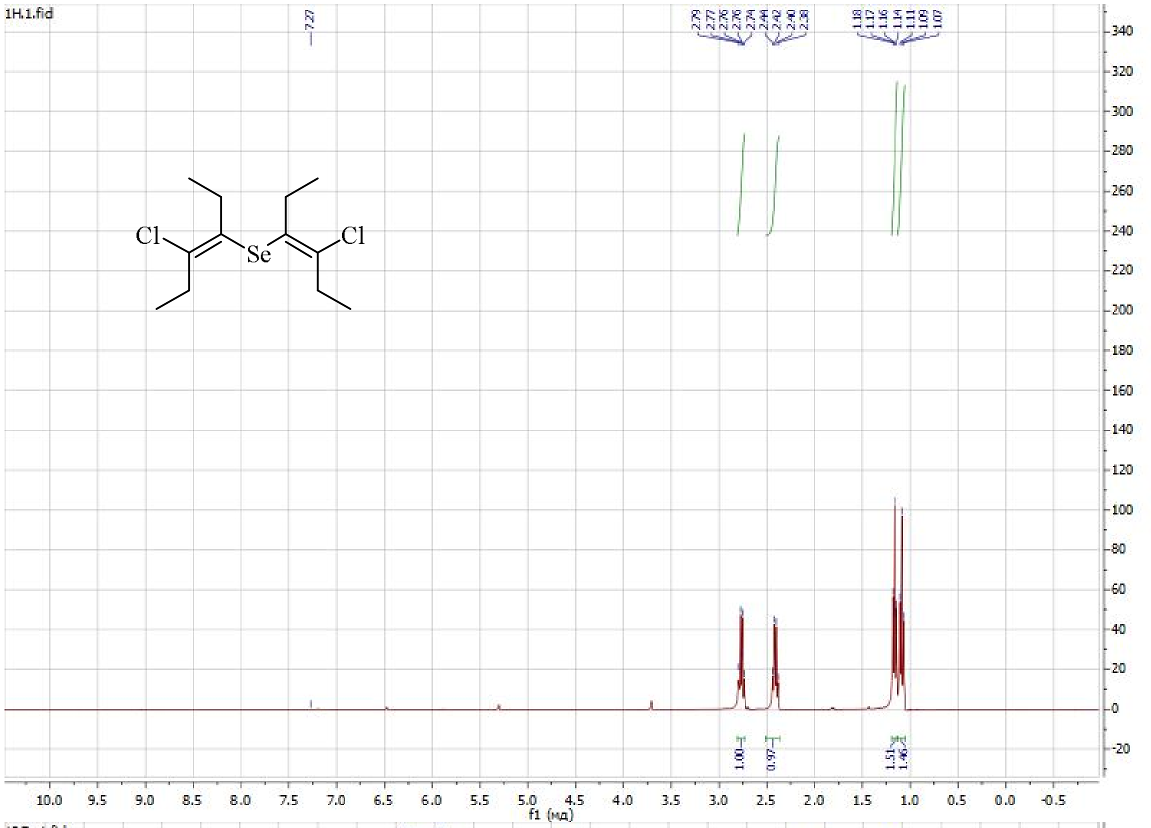

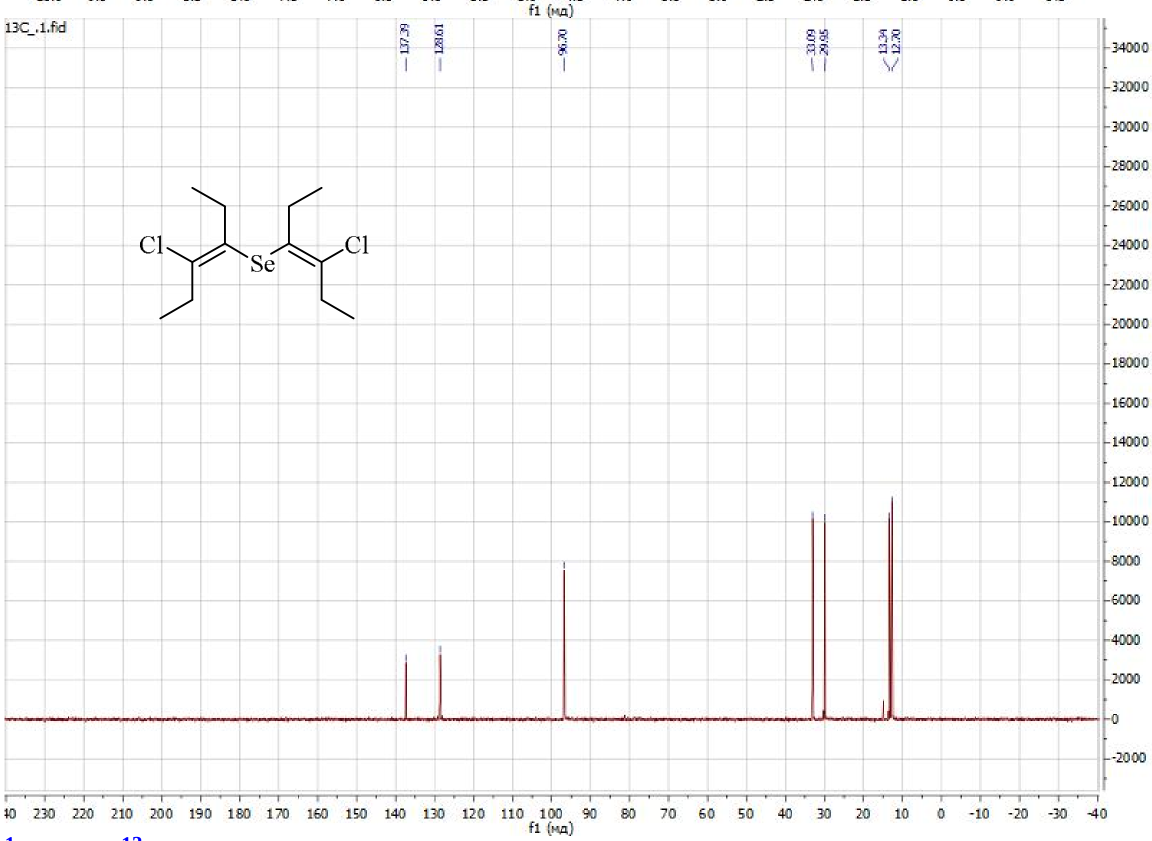

**$^{1}$H- and $^{13}$C-NMR spectra of compound 5**

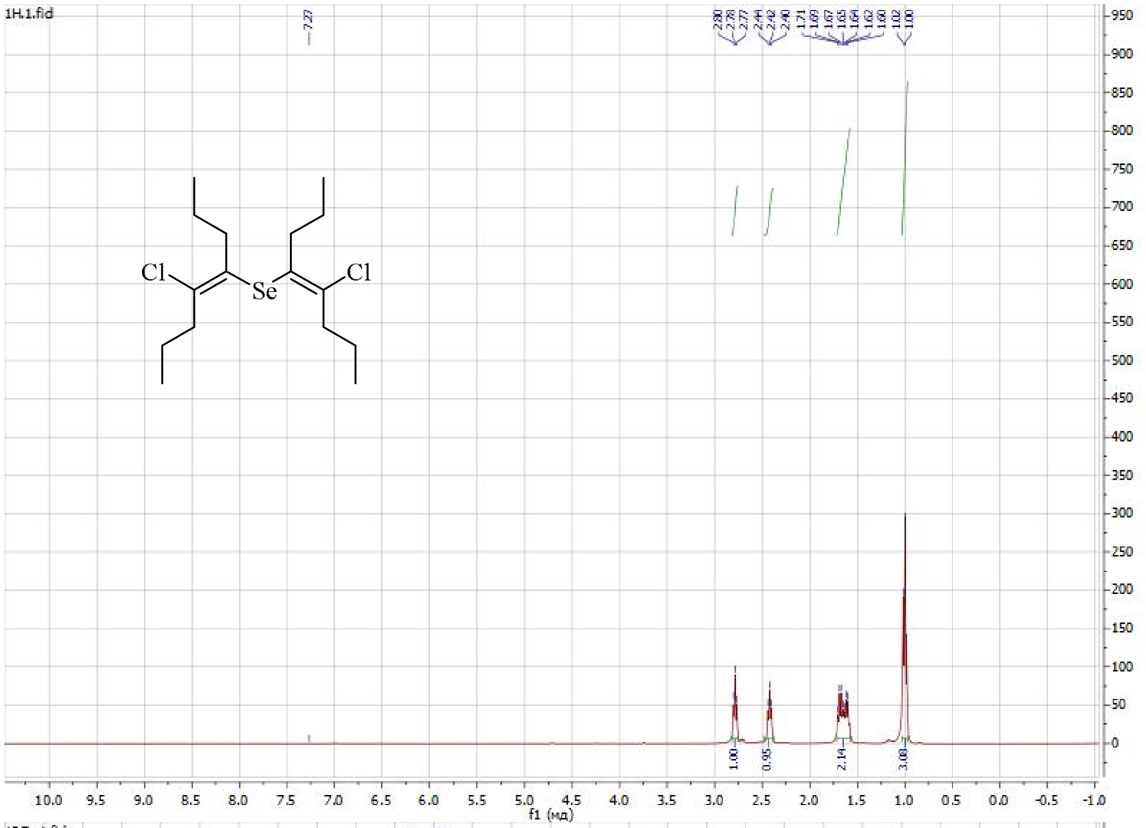

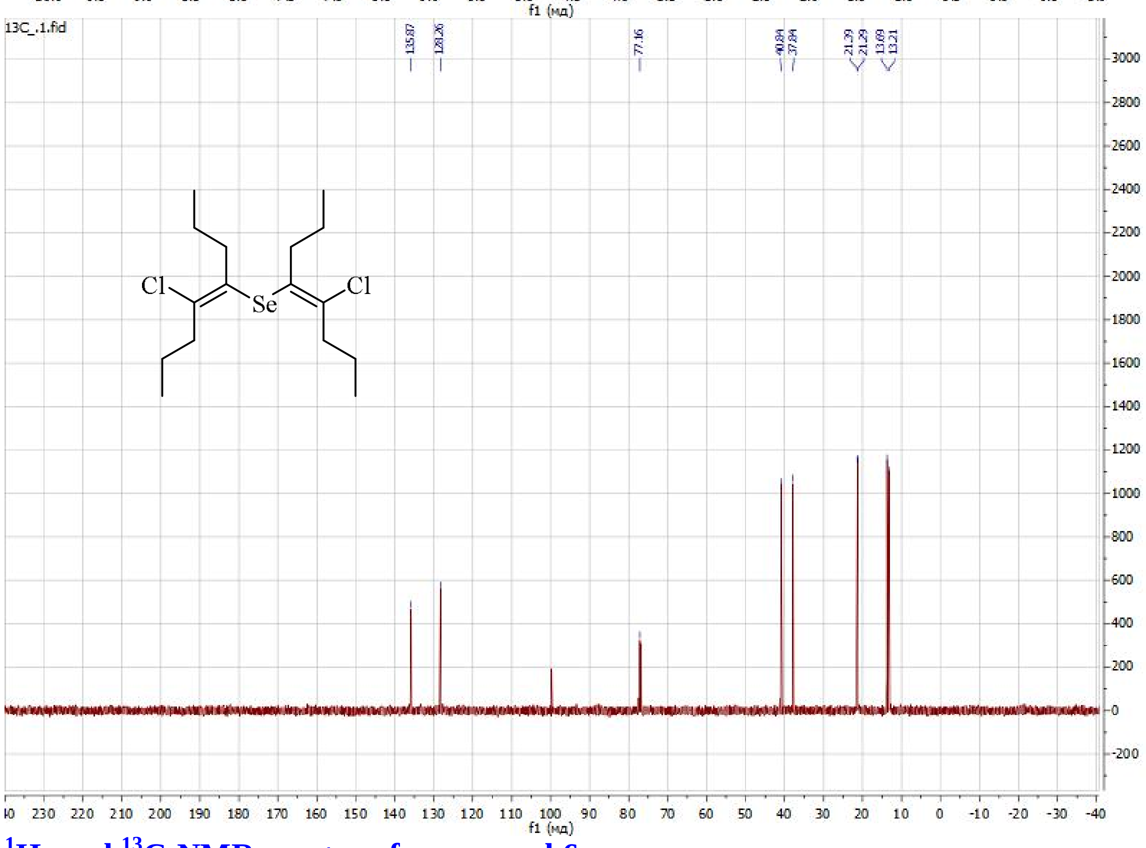

**$^{1}$H- and $^{13}$C-NMR spectra of compound 6**

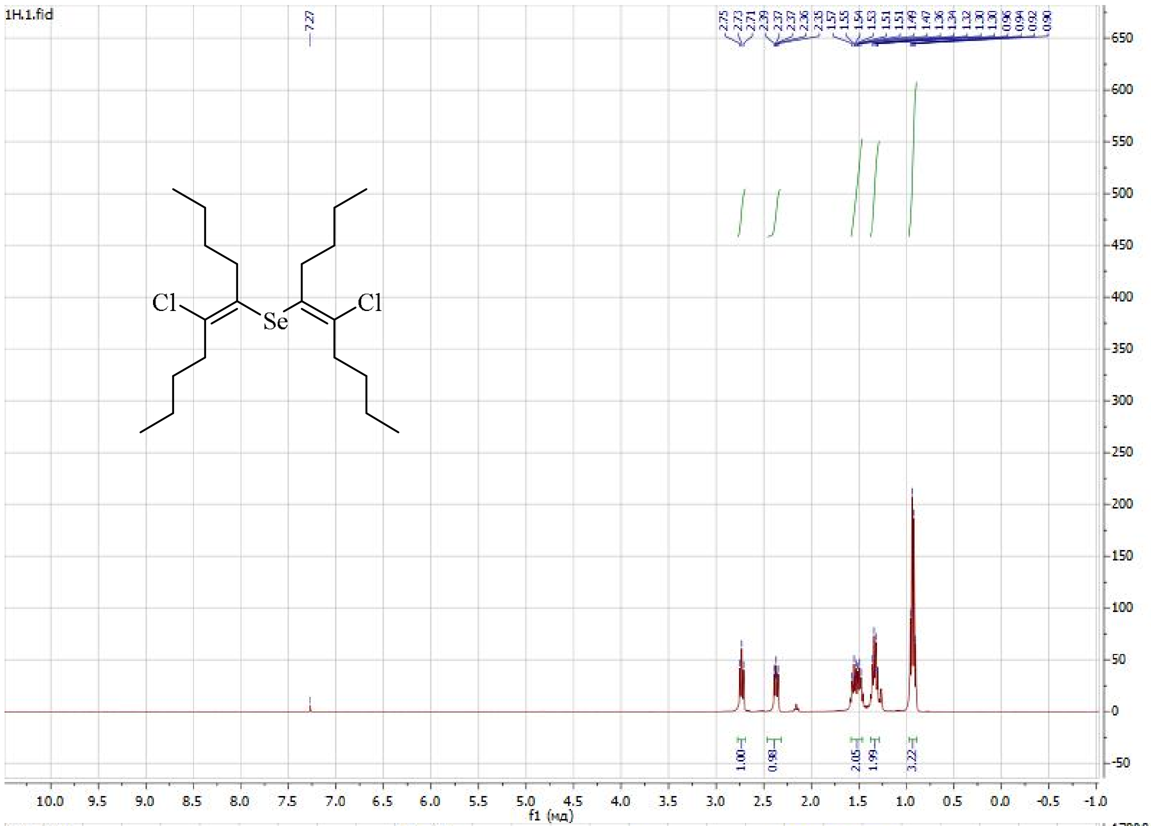


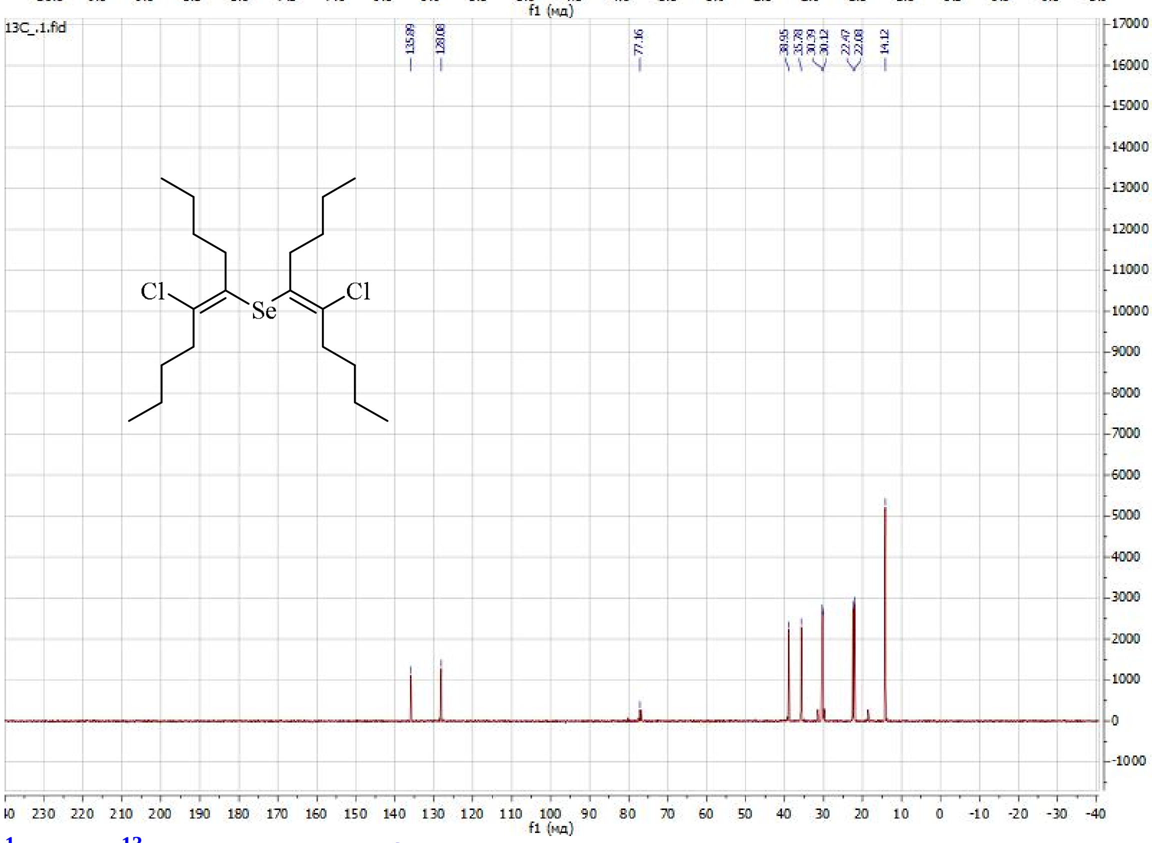


**$^{1}$H- and $^{13}$C-NMR spectra of compound 7**

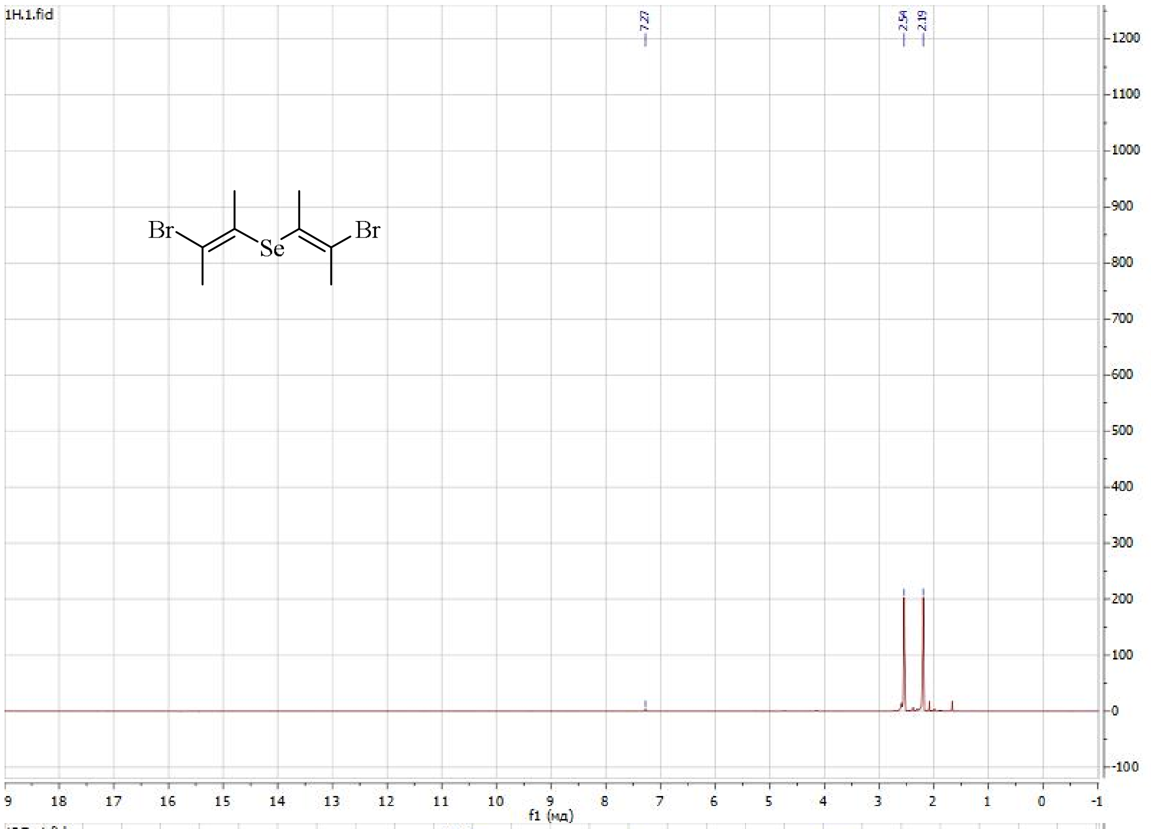


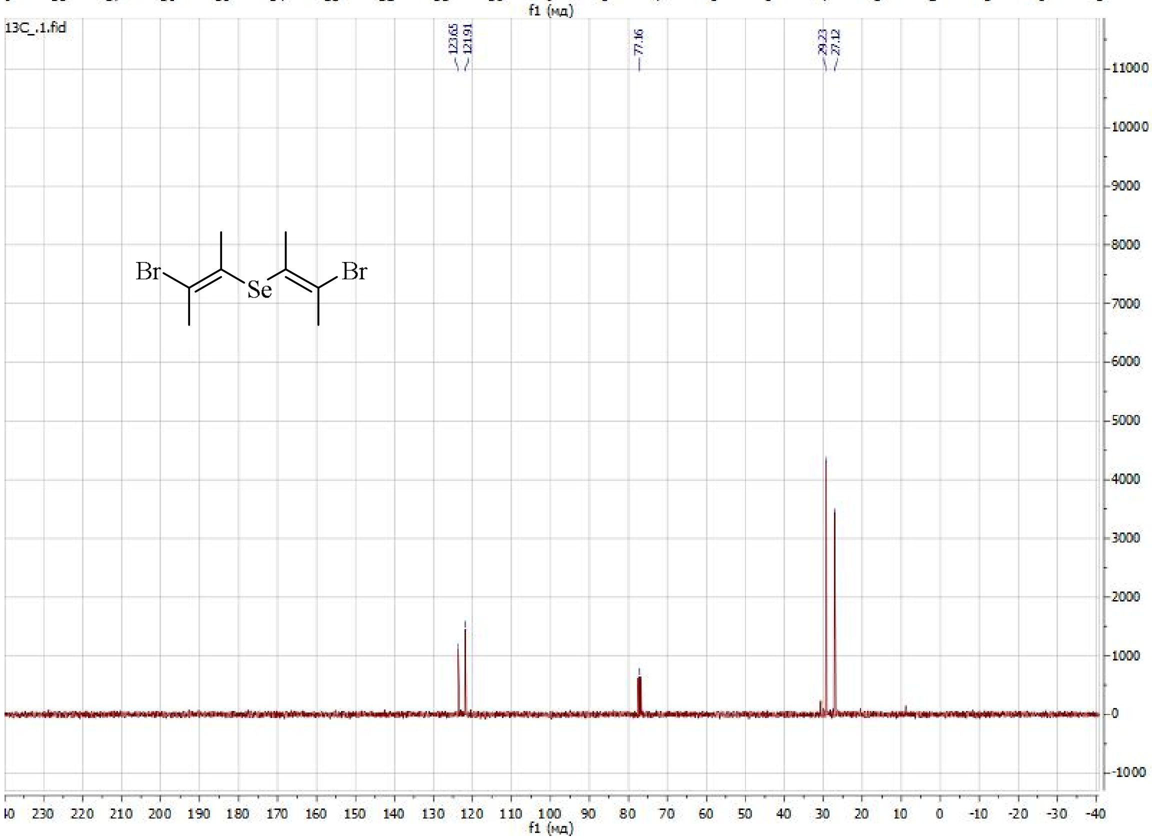


**$^{1}$H- and $^{13}$C-NMR spectra of compound 8**

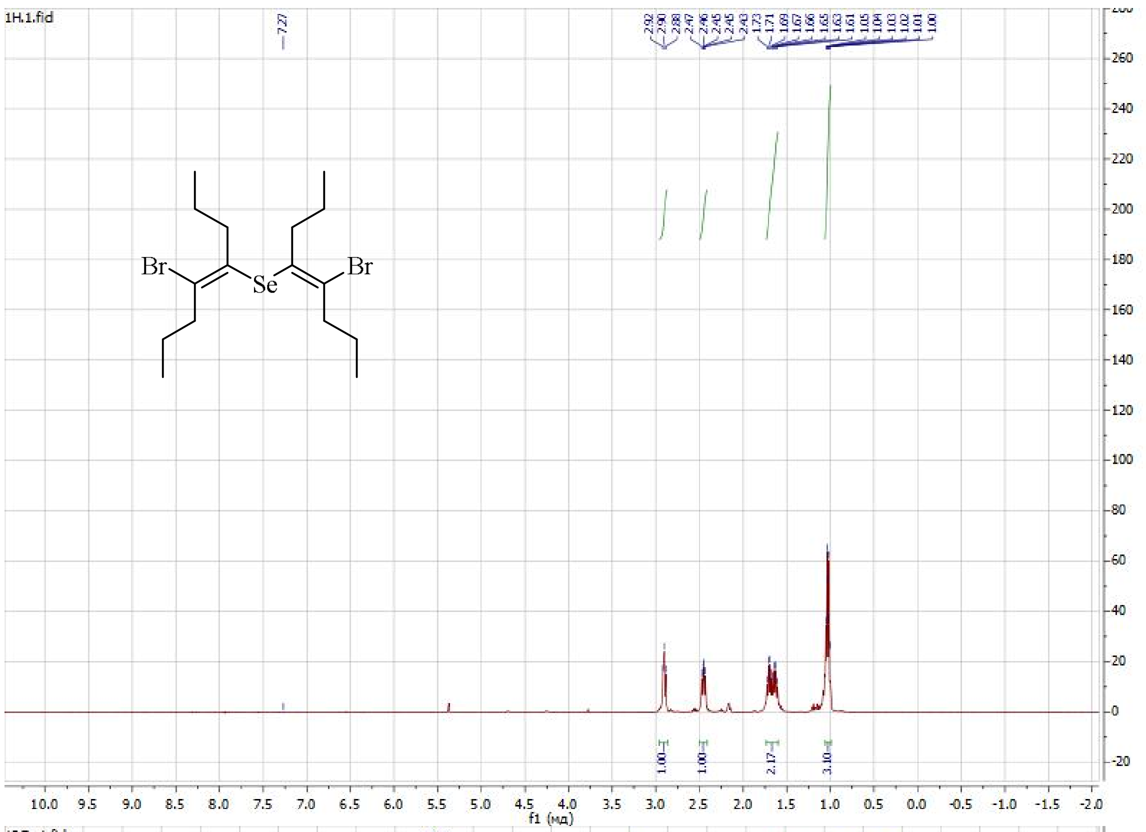

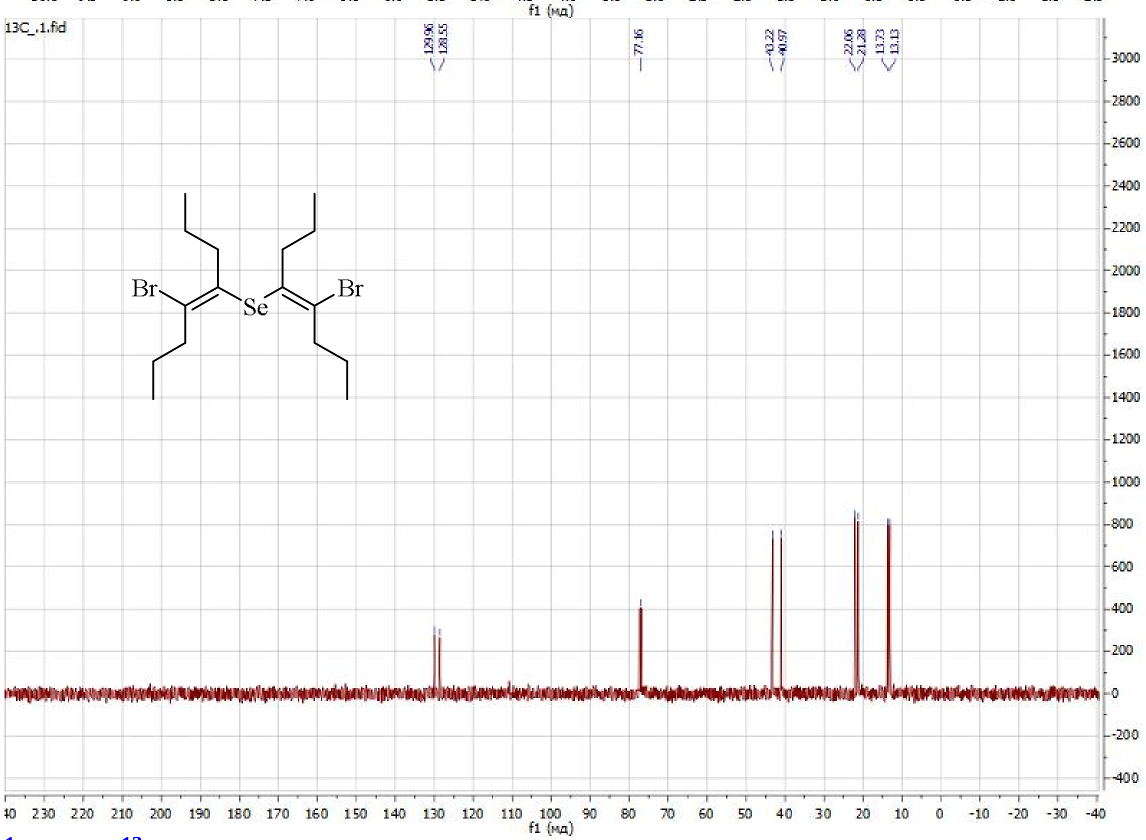

**$^{1}$H- and $^{13}$C-NMR spectra of compound 10**

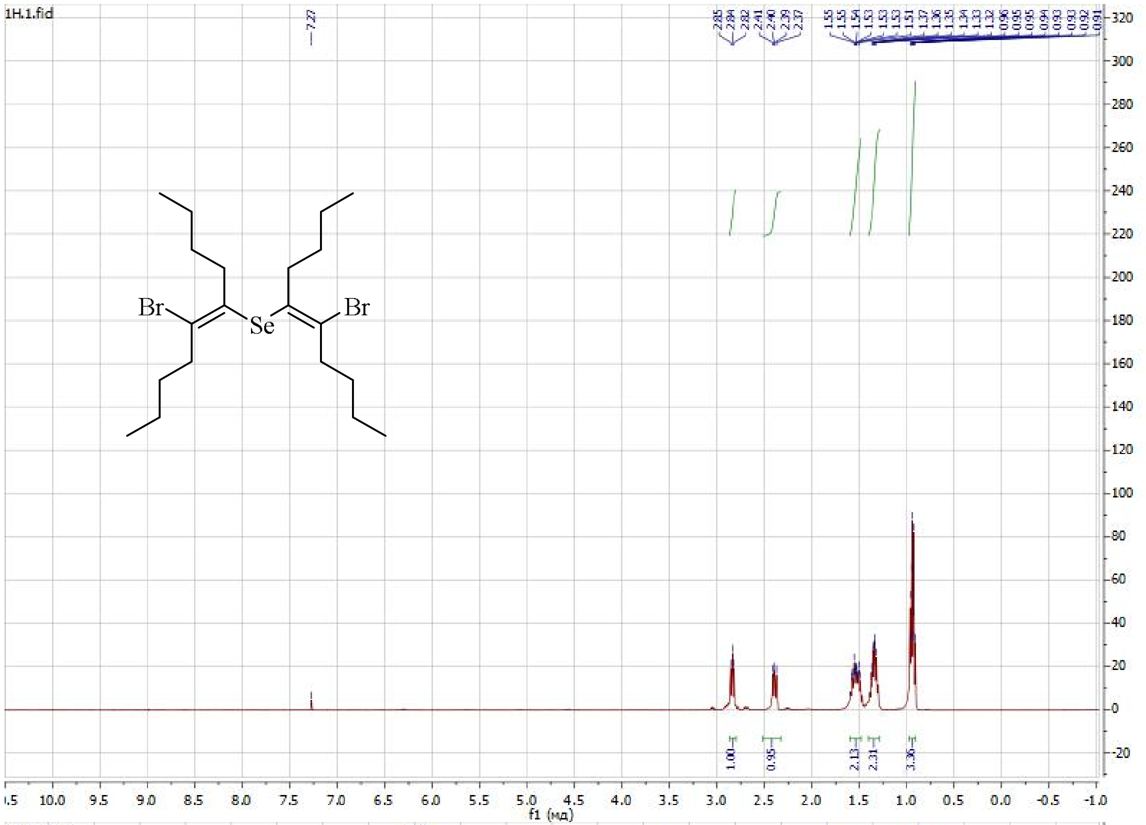


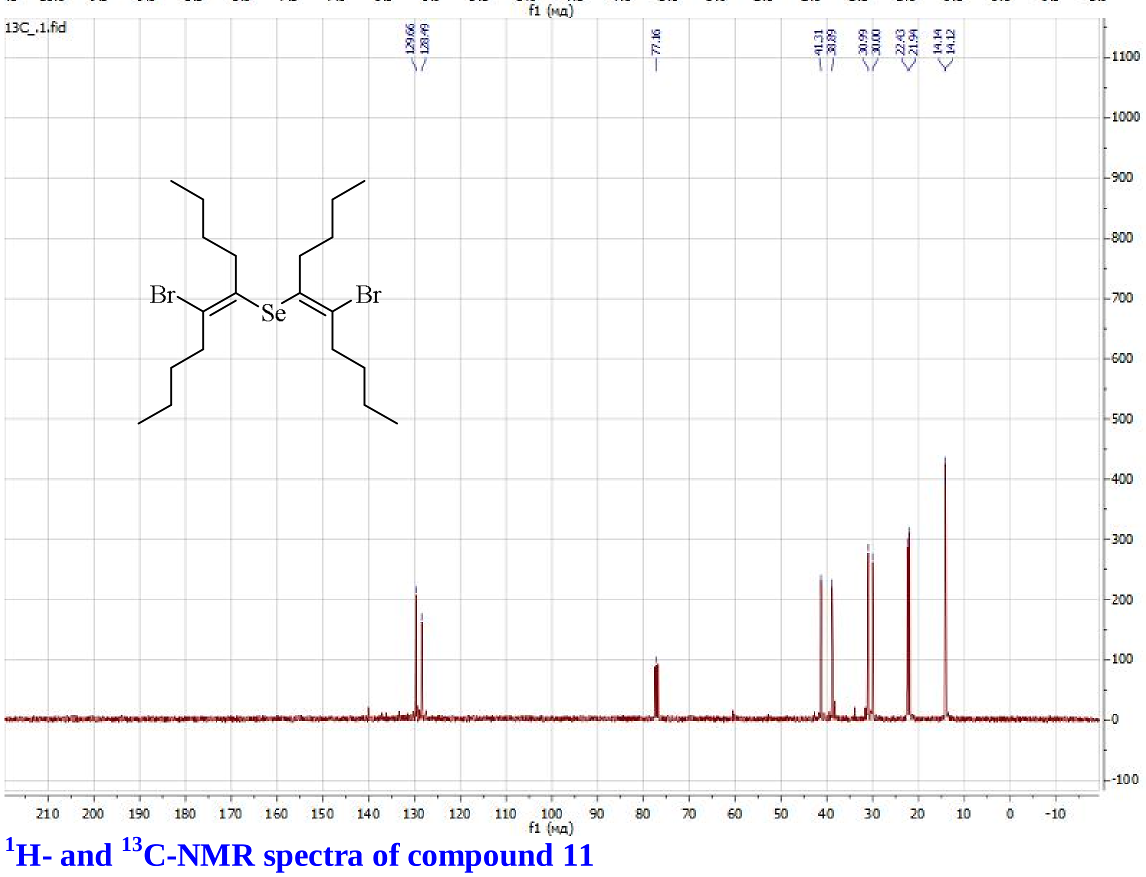


**$^{1}$H- and $^{13}$C-NMR spectra of compound 11**

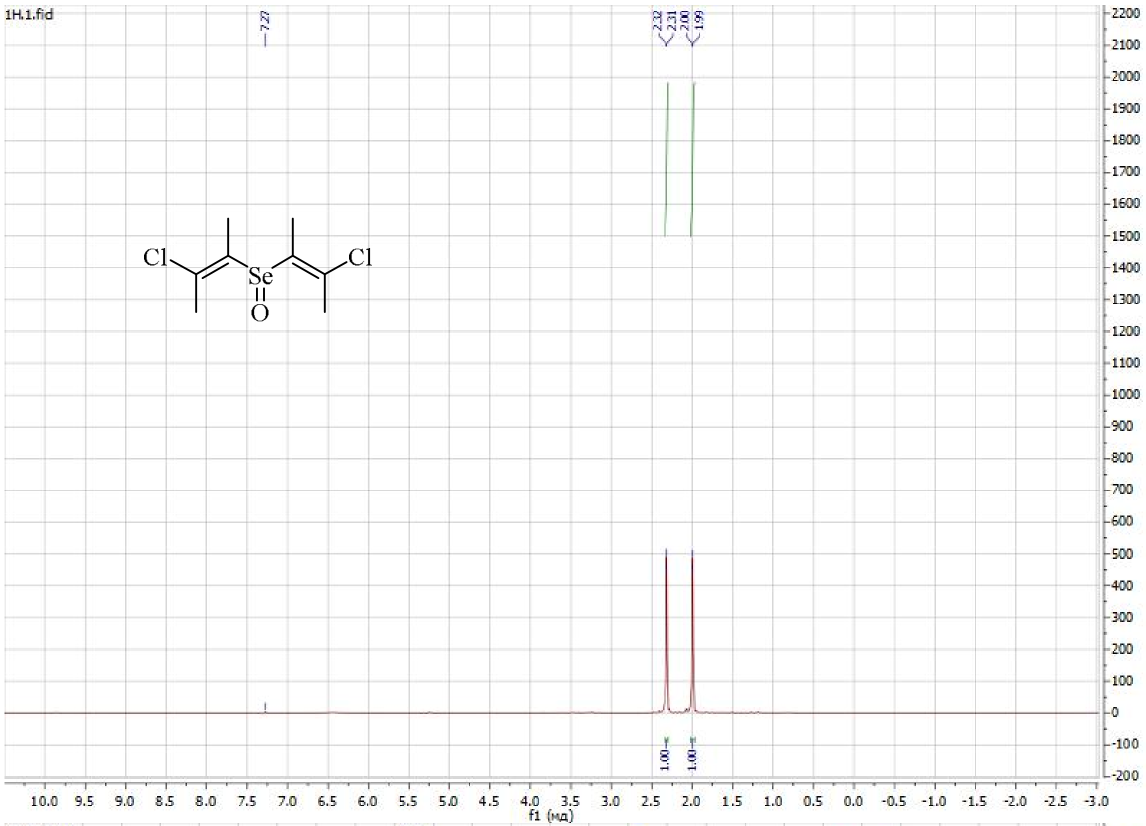


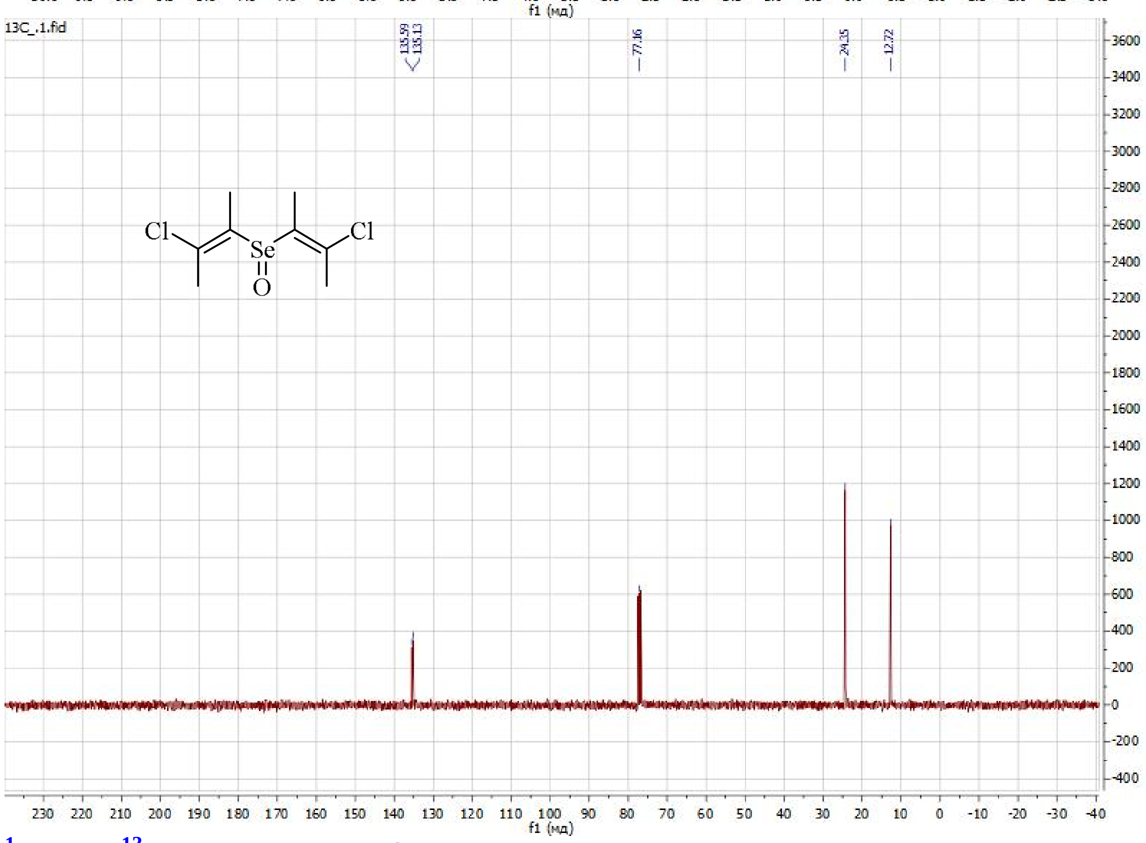


**$^{1}$H- and $^{13}$C-NMR spectra of compound 12**

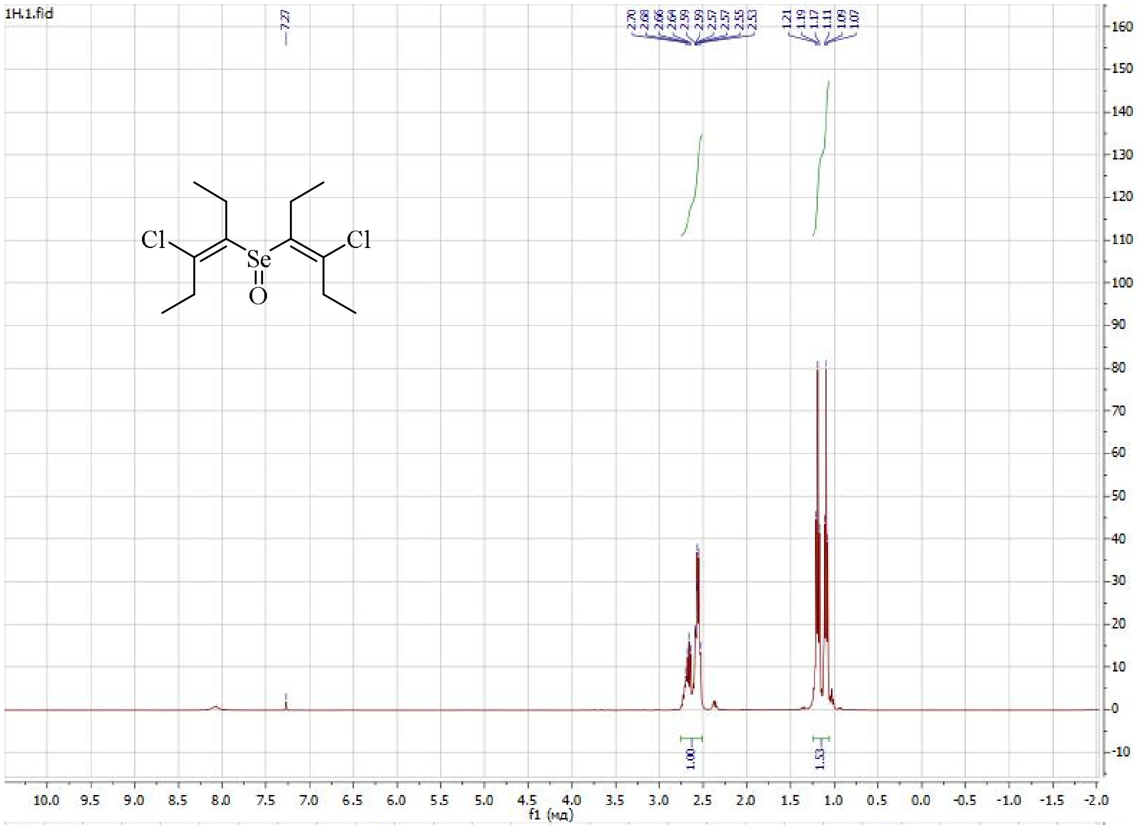


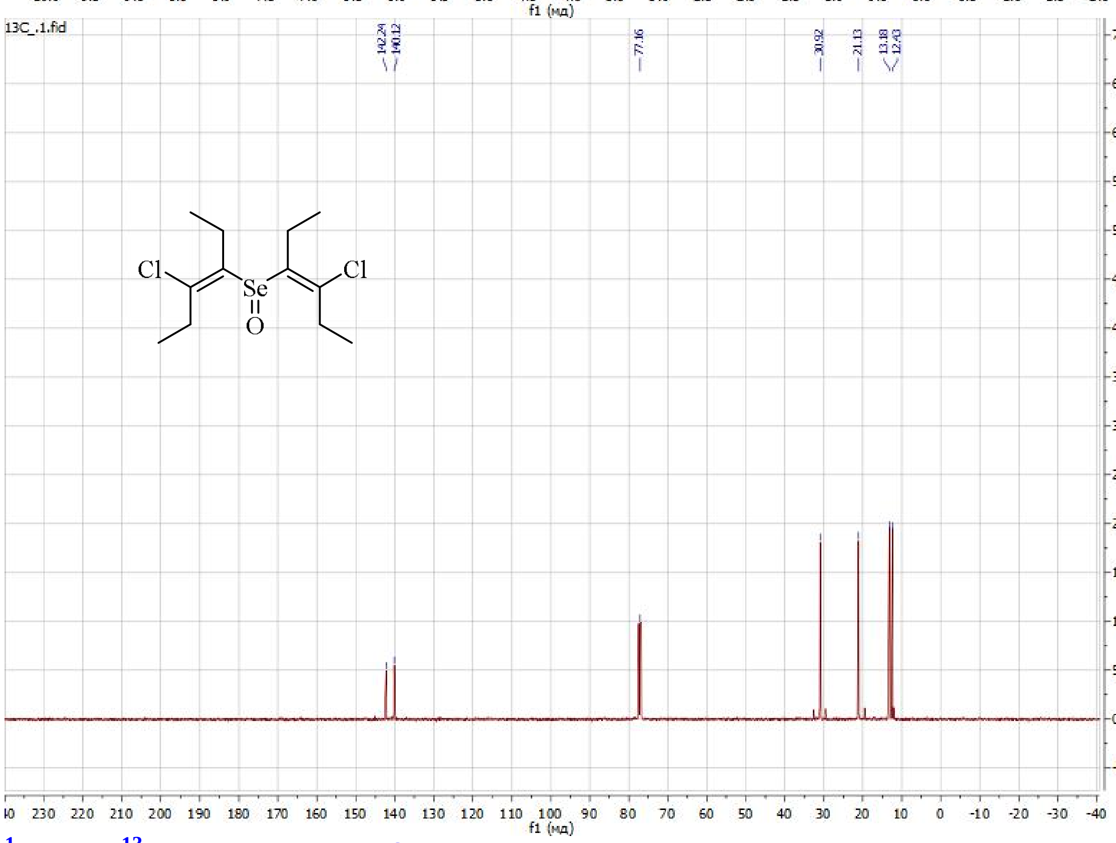


**$^{1}$H- and $^{13}$C-NMR spectra of compound 13**

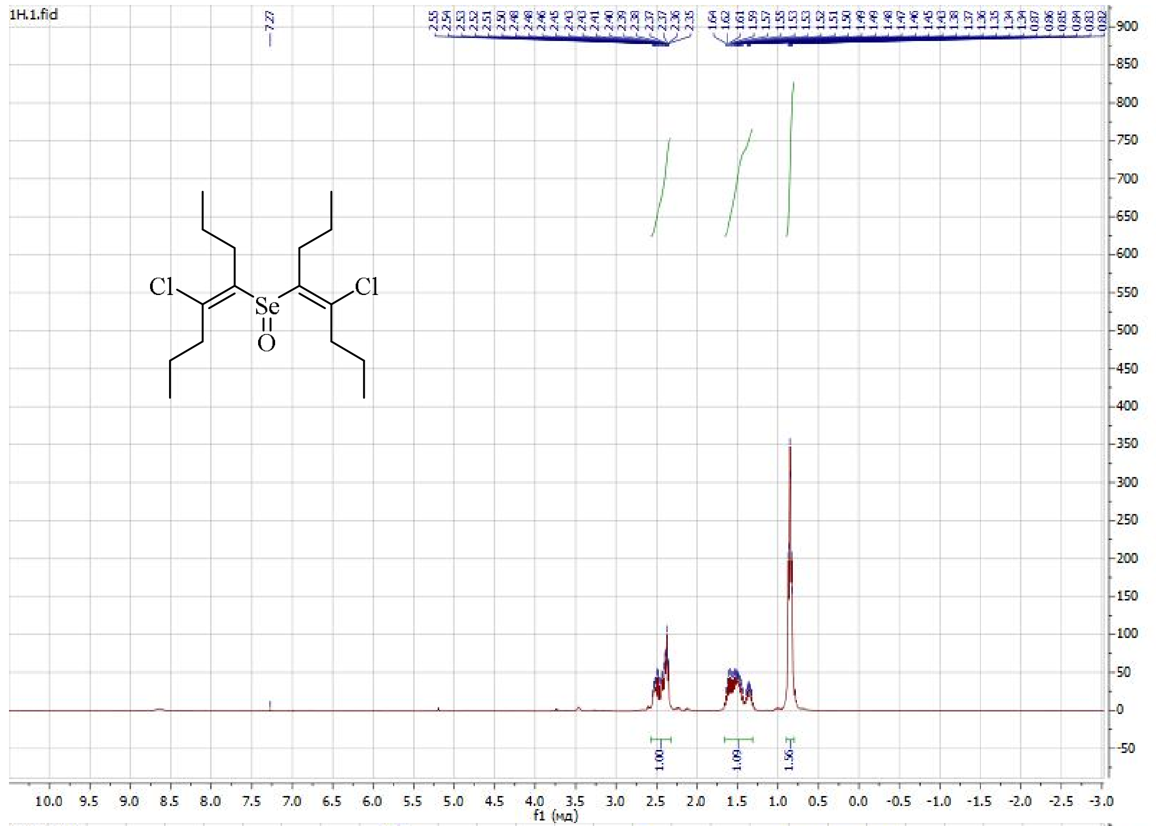

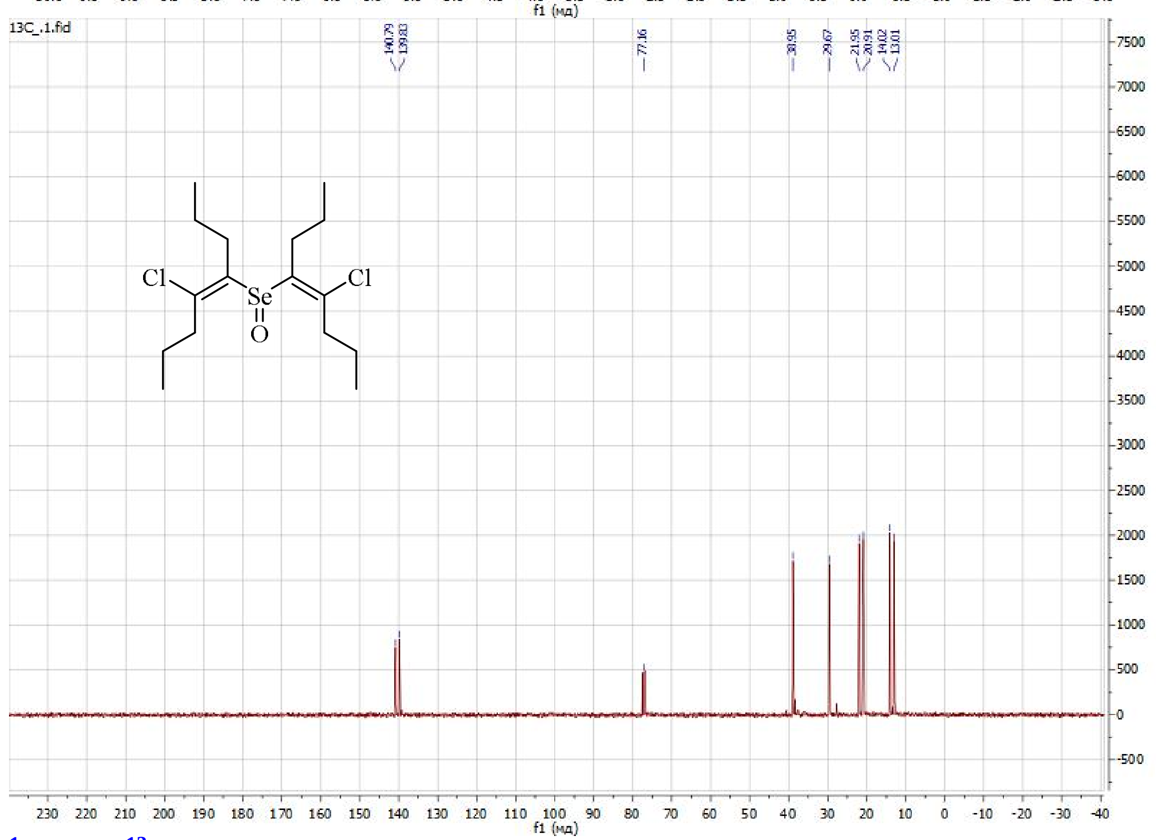

**$^{1}$H- and $^{13}$C-NMR spectra of compound 14**

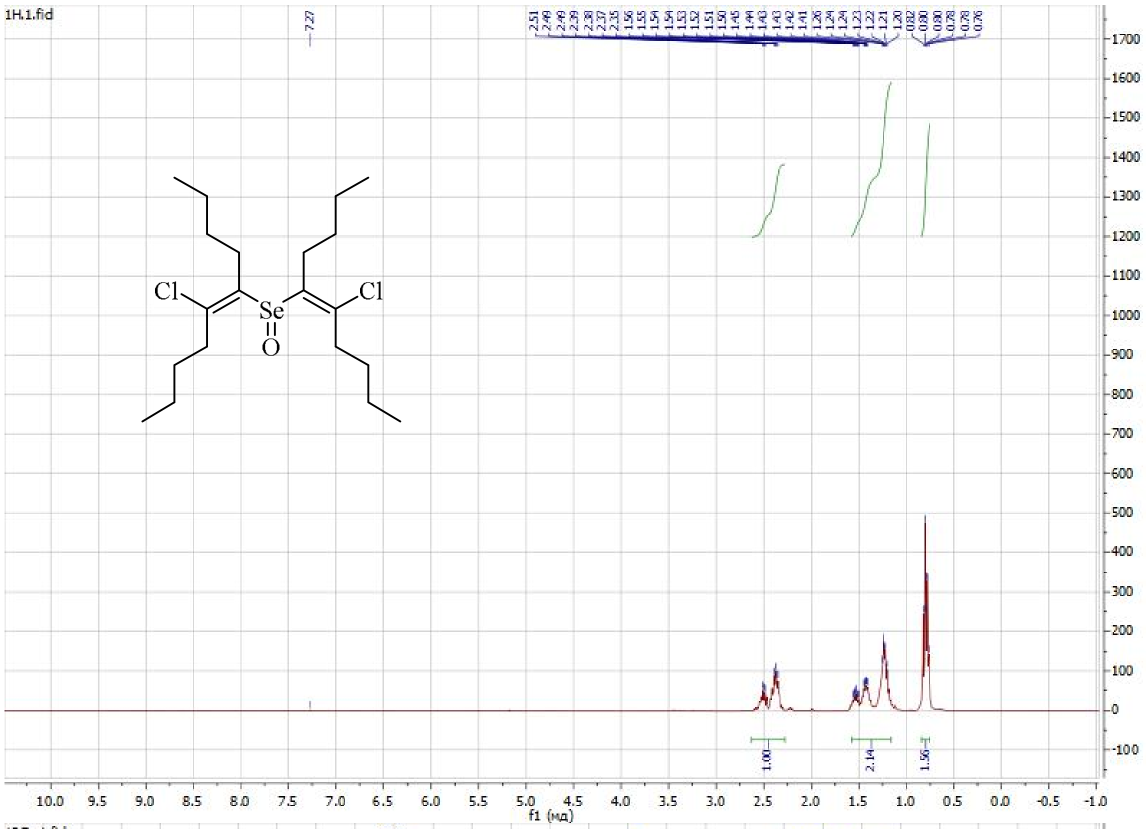

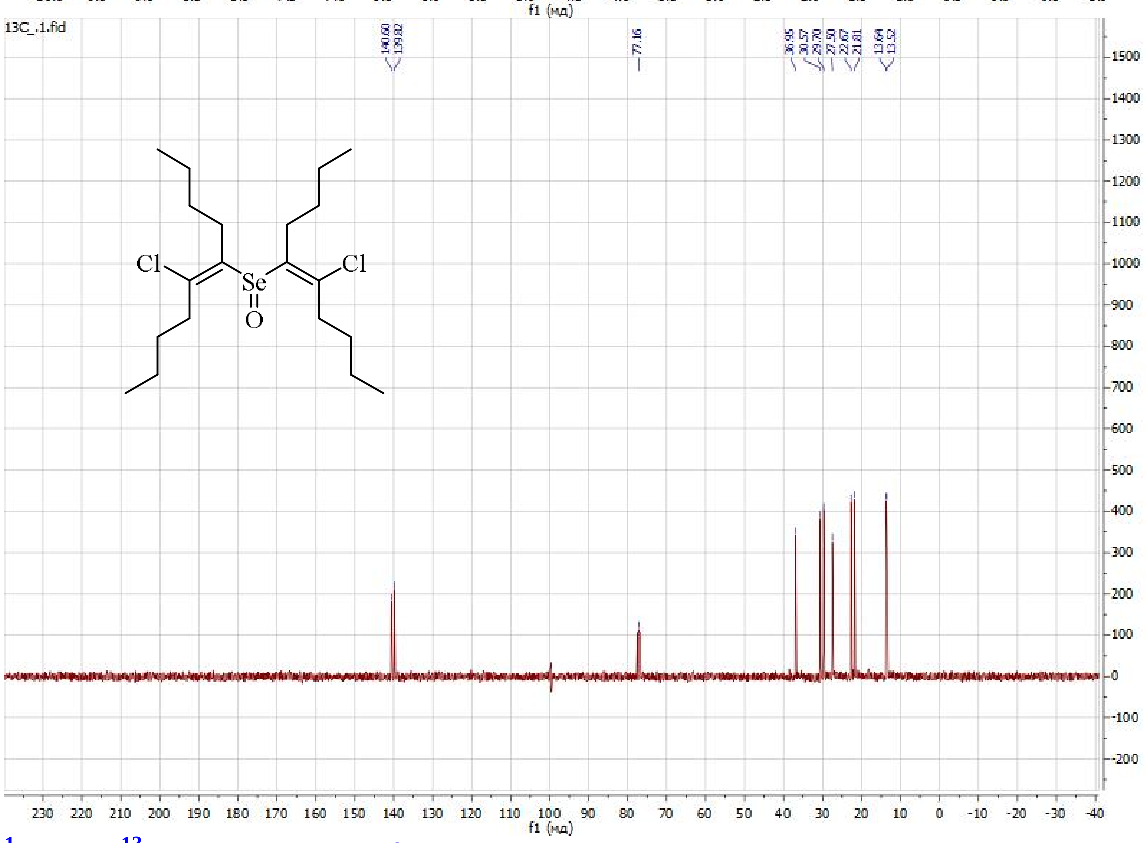

$^{1}$H- and $^{13}$C-NMR spectra of compound 15

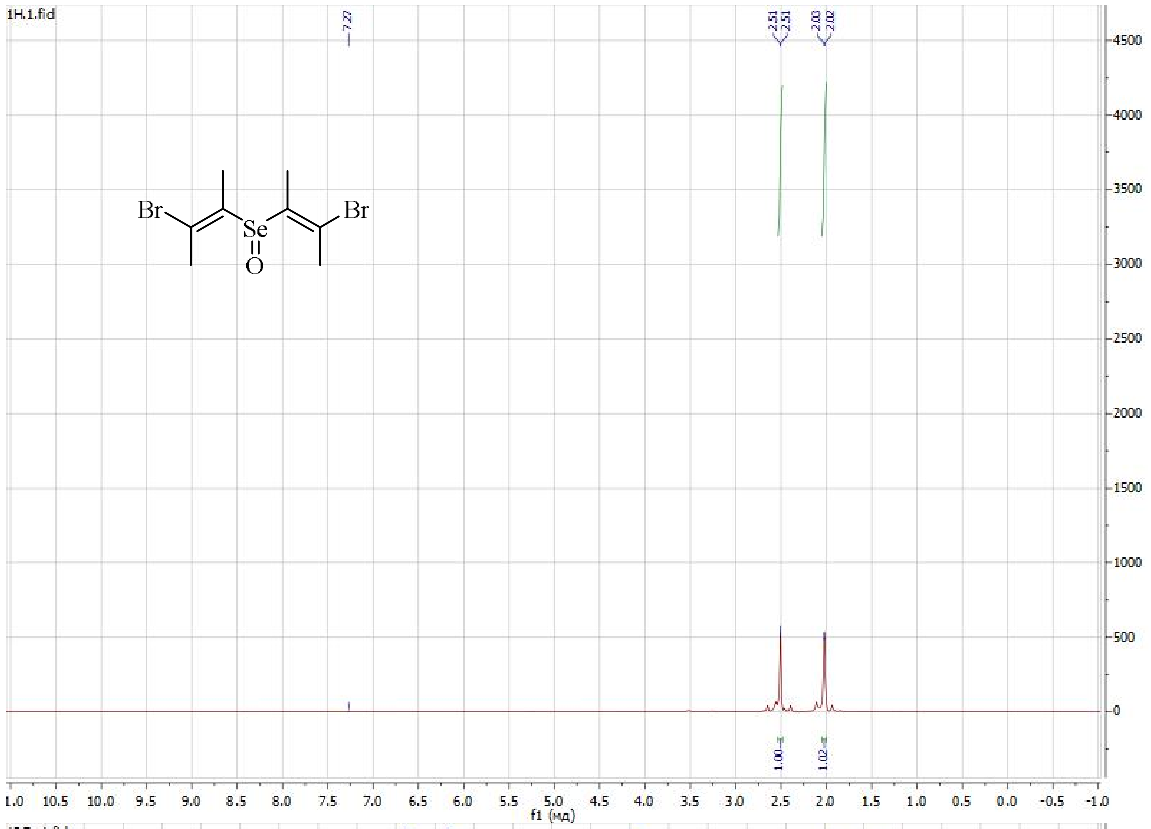


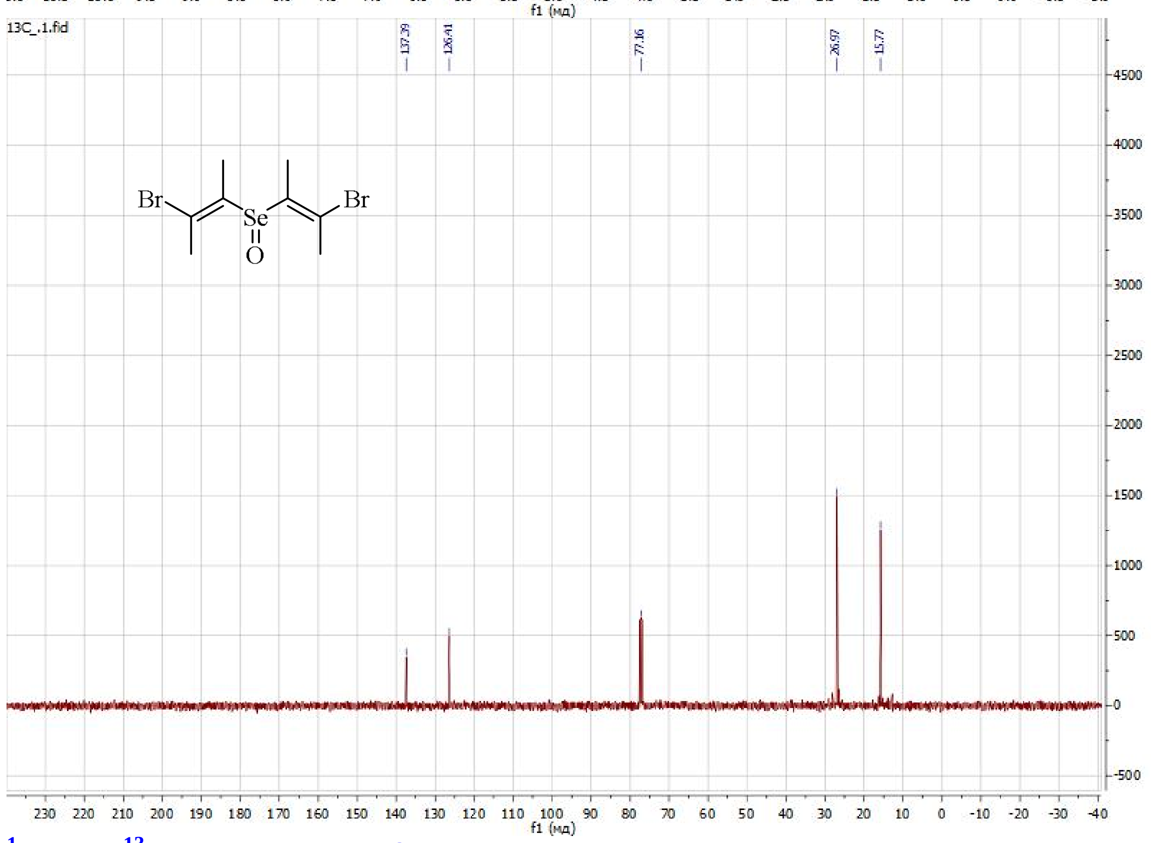


**$^{1}$H- and $^{13}$C-NMR spectra of compound 16**

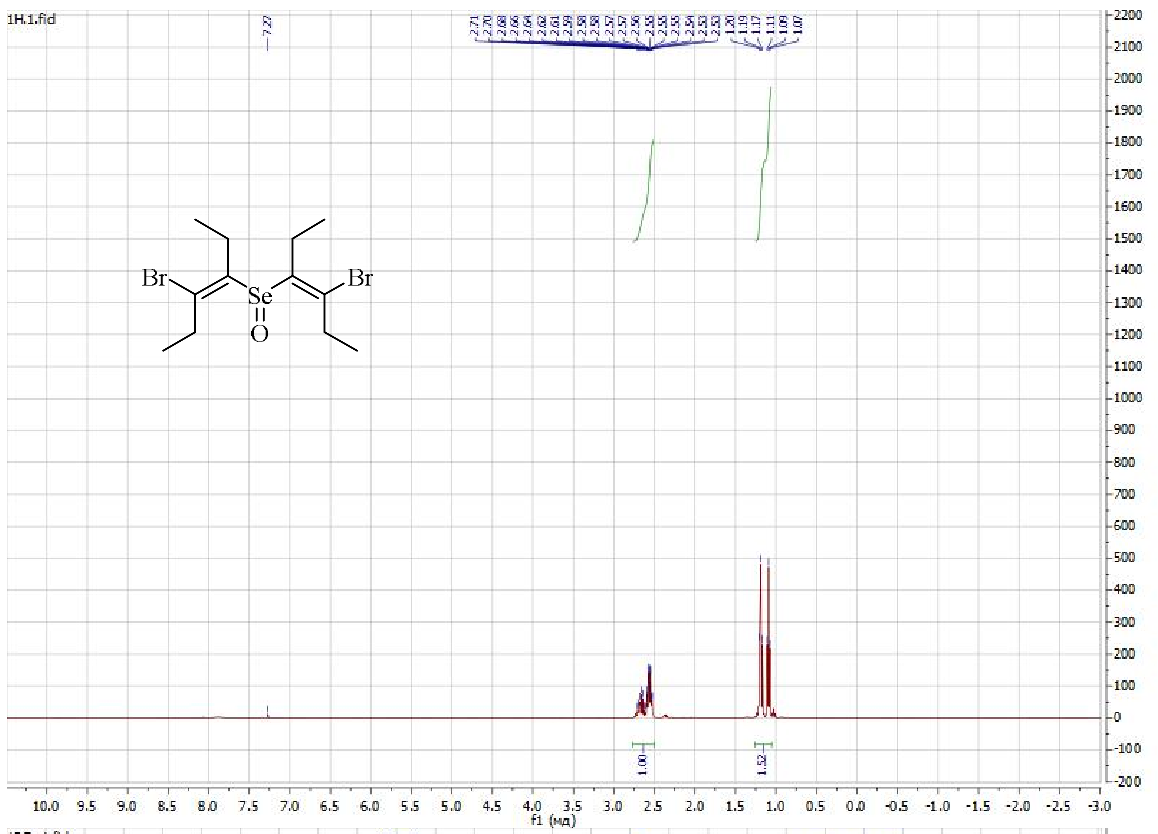


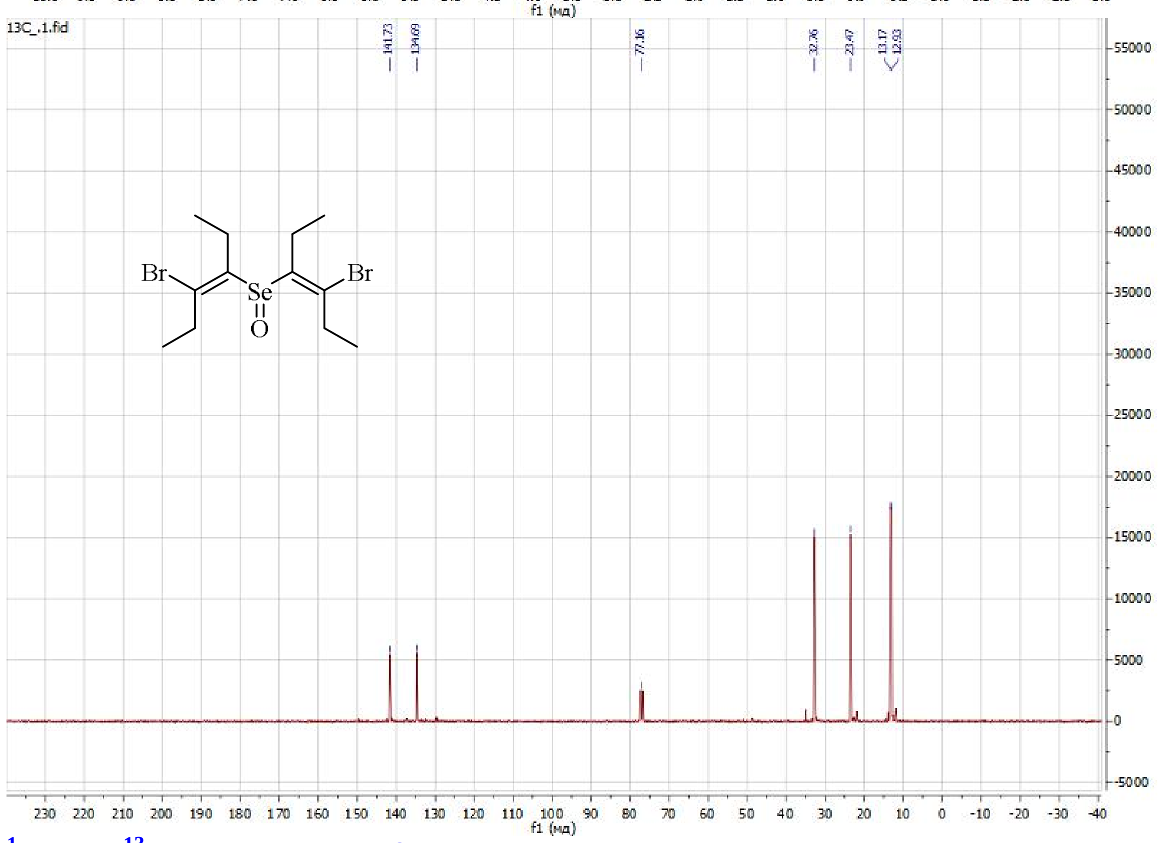


**$^{1}$H- and $^{13}$C-NMR spectra of compound 17**

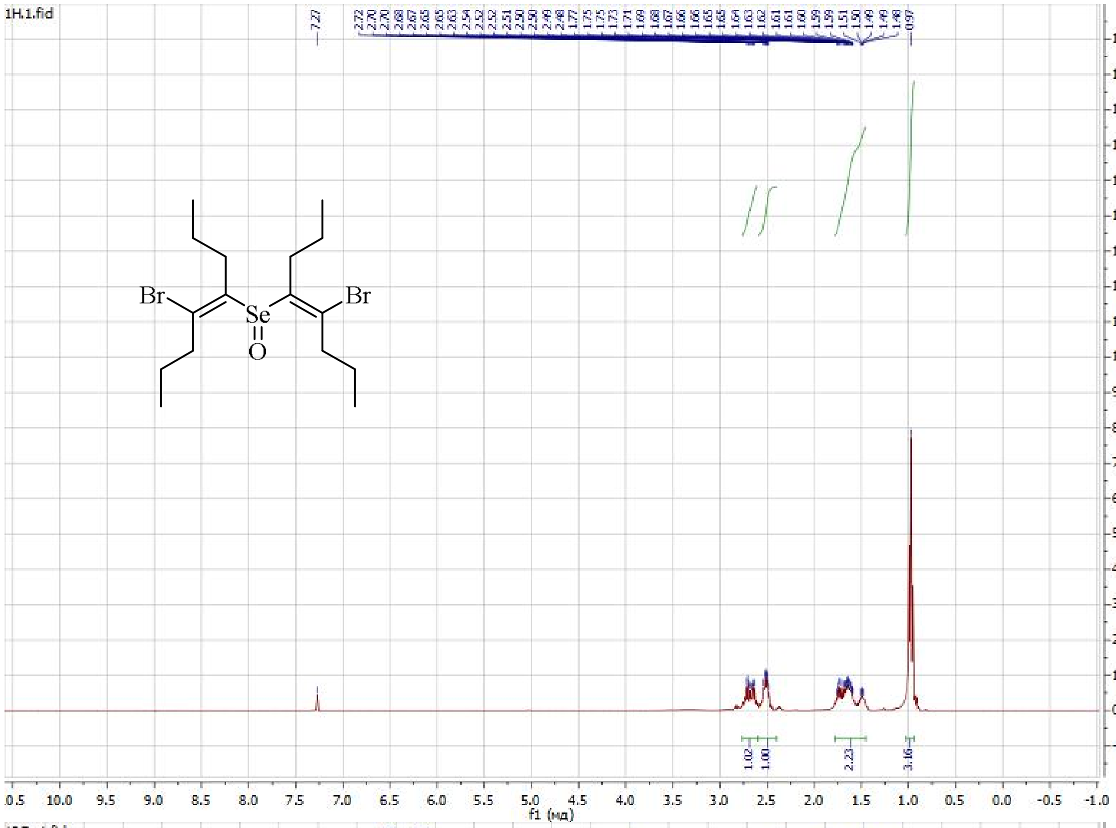

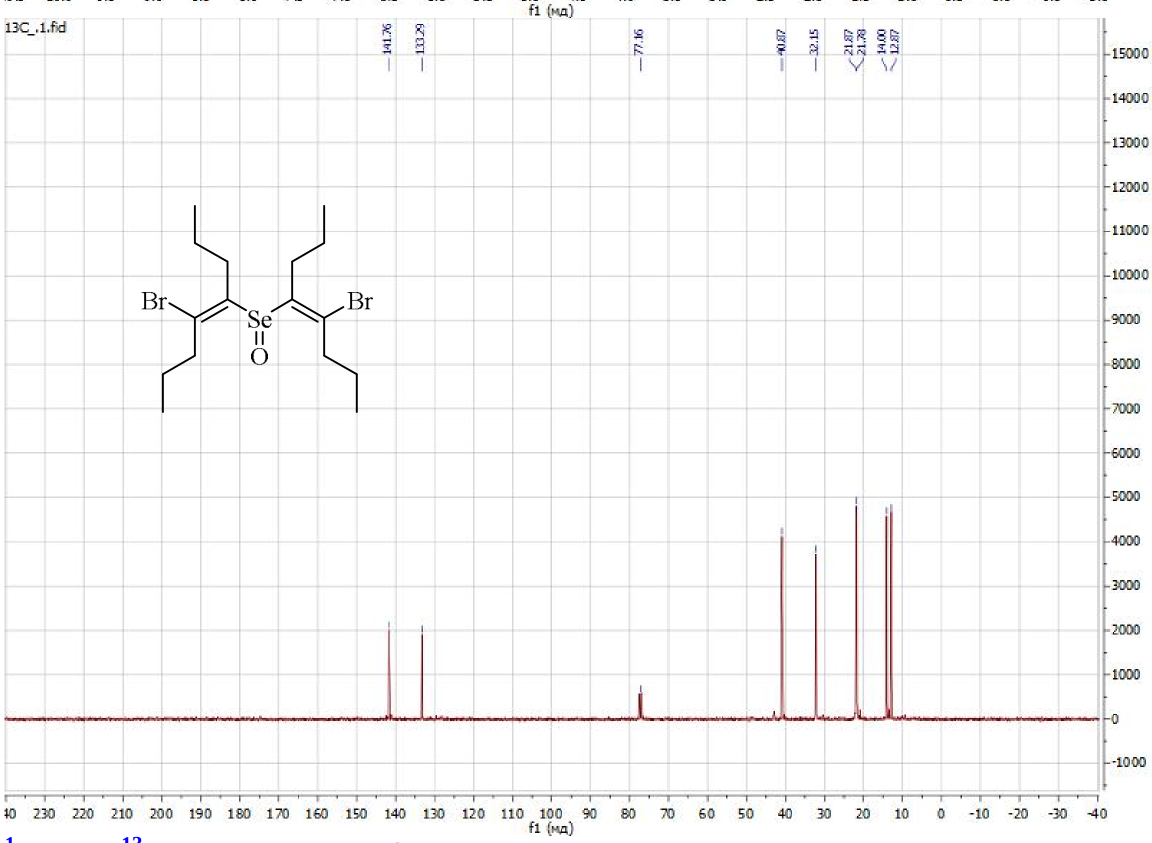

**$^{1}$H- and $^{13}$C-NMR spectra of compound 18**

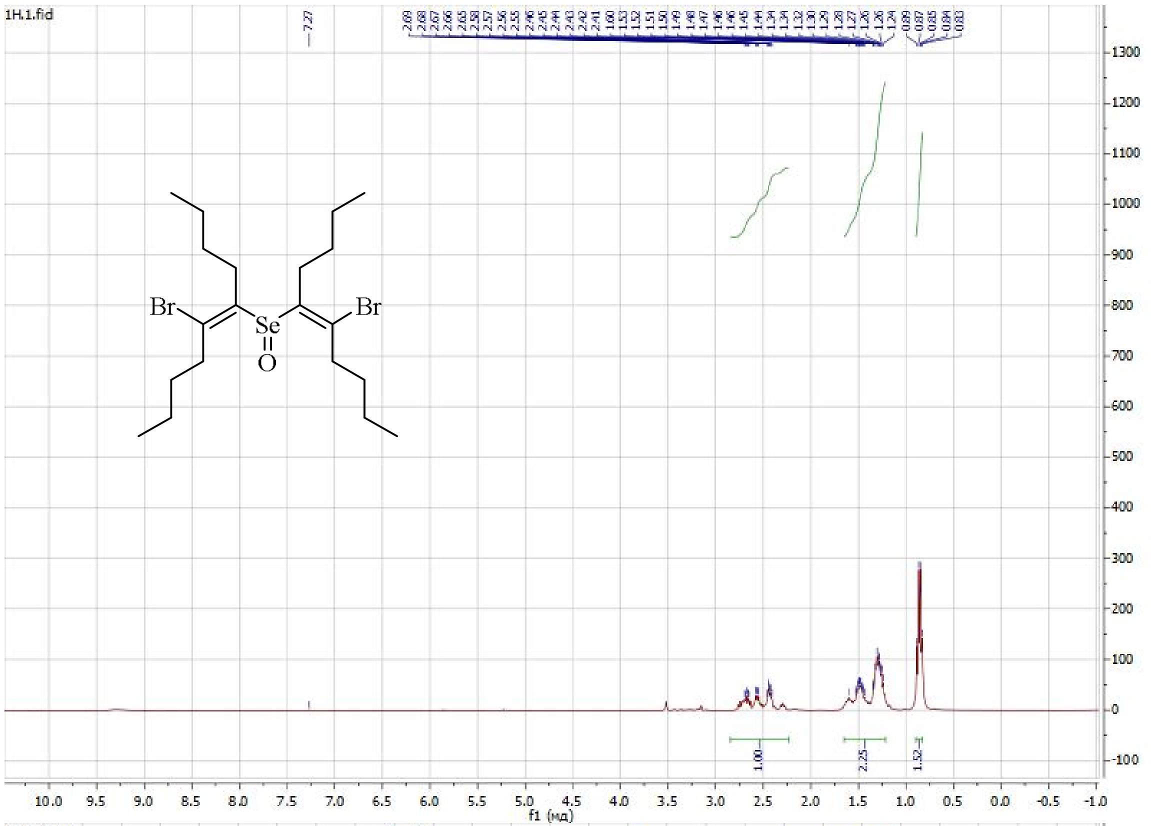


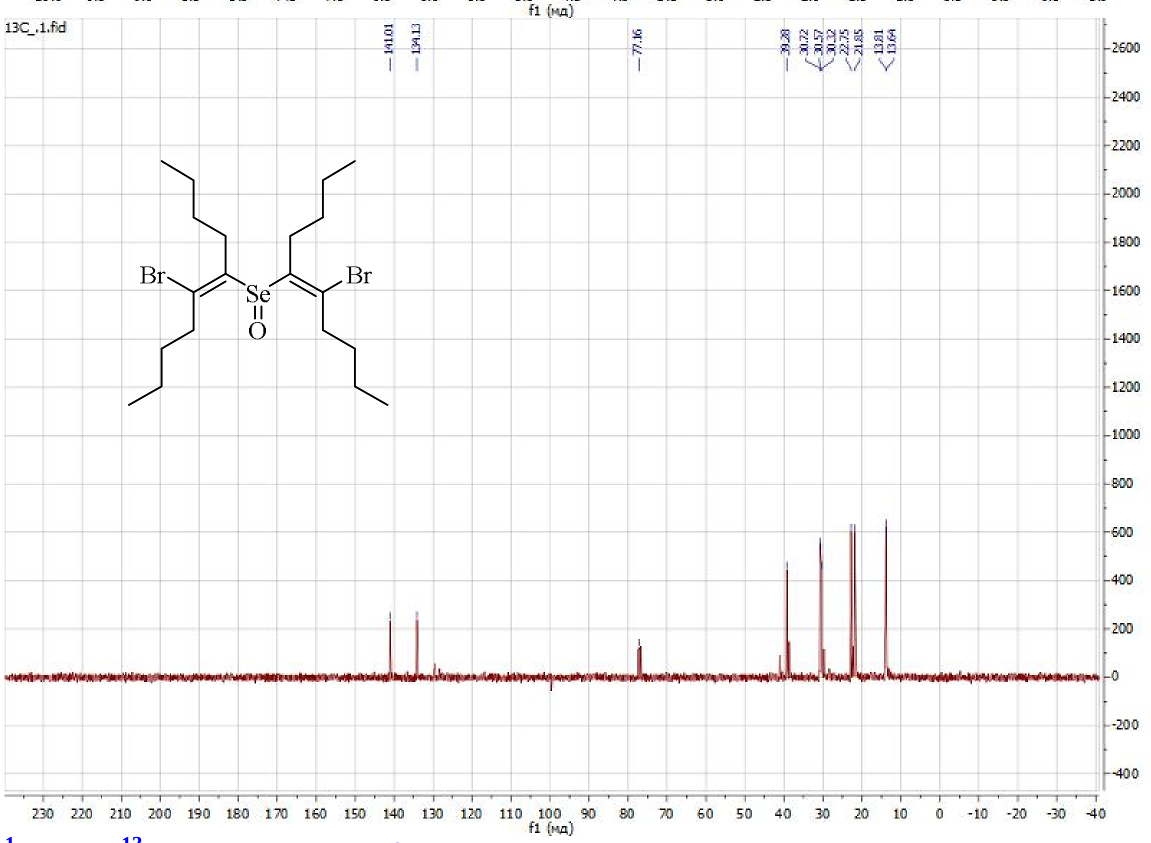


**$^{1}$H- and $^{13}$C-NMR spectra of compound 19**
